# Characterization of genetic diversity in Turkish common bean gene pool using phenotypic and whole-genome DArTseq-generated silicoDArT marker information

**DOI:** 10.1371/journal.pone.0205363

**Published:** 2018-10-11

**Authors:** Muhammad Azhar Nadeem, Ephrem Habyarimana, Vahdettin Çiftçi, Muhammad Amjad Nawaz, Tolga Karaköy, Gonul Comertpay, Muhammad Qasim Shahid, Rüştü Hatipoğlu, Mehmet Zahit Yeken, Fawad Ali, Sezai Ercişli, Gyuhwa Chung, Faheem Shehzad Baloch

**Affiliations:** 1 Department of field crops, Faculty of Agricultural and Natural Science, Abant Izzet Baysal University, Bolu, Turkey; 2 Consiglio per la ricerca in agricoltura e l'analisi dell'economia agraria–Centro di ricerca cerealicoltura e colture industriali, Bologna, Italy; 3 Department of Biotechnology, Chonnam National University, Chonnam, Republic of Korea; 4 Organic Agriculture Program, Vocational School of Sivas, University of Cumhuriyet, Sivas, Turkey; 5 Eastern Mediterranean Agricultural Research Institute, Turkey; 6 State Key Laboratory for Conservation and Utilization of Subtropical Agro-bio resources, South China Agricultural University, Guangzhou, China; 7 Department of Field Crops, Faculty of Agricultural, University of Cukurova, Adana, Turkey; 8 Department of Horticulture, Faculty of Agriculture, Ataturk University, Erzurum, Turkey; Chinese Academy of Sciences, CHINA

## Abstract

Turkey presents a great diversity of common bean landraces in farmers’ fields. We collected 183 common bean accessions from 19 different Turkish geographic regions and 5 scarlet runner bean accessions to investigate their genetic diversity and population structure using phenotypic information (growth habit, and seed weight, flower color, bracteole shape and size, pod shape and leaf shape and color), geographic provenance and 12,557 silicoDArT markers. A total of 24.14% markers were found novel. For the entire population (188 accessions), the expected heterozygosity was 0.078 and overall gene diversity, Fst and Fis were 0.14, 0.55 and 1, respectively. Using marker information, model-based structure, principal coordinate analysis (PCoA) and unweighted pair-group method with arithmetic means (UPGMA) algorithms clustered the 188 accessions into two main populations A (predominant) and B, and 5 unclassified genotypes, representing 3 meaningful heterotic groups for breeding purposes. Phenotypic information clearly distinguished these populations; population A and B, respectively, were bigger (>40g/100 seeds) and smaller (<40g/100 seeds) seed-sized. The unclassified population was pure and only contained climbing genotypes with 100 seed weight 2–3 times greater than populations A and B. Clustering was mainly based on A: seed weight, B: growth habit, C: geographical provinces and D: flower color. Mean kinship was generally low, but population B was more diverse than population A. Overall, a useful level of gene and genotypic diversity was observed in this work and can be used by the scientific community in breeding efforts to develop superior common bean strains.

## 1. Introduction

Common bean (*Phaseolus vulgaris* L.) is one of the most ancient grain legumes of America [1–2–3], and is an important proteinaceous staple food for more than 300 million people [4]. It is a self-pollinated crop with a small genome size of 587 Mbs [5]. The genus *Phaseolus* contains more than 70 species five of which (*P*. *vulgaris* L., *P*. *lunatus* L., *P*. *acutifolius* A. Gray., *P*. *coccineus* L. and *P*. *dumosus*) being the most cultivated [6]. *P*. *coccineus* commonly known as scarlet runner bean is close relative of common [7] and 3^rd^ most economically important bean species after common bean and *P*. *lunatus* [8]. Runner bean is a climbing perennial crop but often grown as annual crop for green pod production [6].

Geographical origin of common bean remained unresolved until Bitocchi et al. [9] proposed the Mesoamerica as the center of origin on the basis of five loci analysis. Domestication is a very complex and important process which involves the modification of wild plants into a crop [10]. Some scientists found multiple domestication events in the common bean [11–12–13–14], while others such as Kwak and Gepts [15] and Rossi et al. [16] were in the favor of a single domestication event. Domestication of common bean resulted in the formation of two diverse gene pools i.e. Mesoamerican and Andean gene pools [17]. Andean gene pool extends from Southern Peru to Northwestern Argentina, while Mesoamerican gene pool extends between Colombia and Northern Mexico [15]. Andean gene pool contains three races i.e., Peru, Nueva Granada, and Chile, while Mesoamerica, Durango, and Jalisco are the three Mesoamerican gene pool races [18].

Europe is considered as secondary diversification center of common bean, and the introduction of this species into Europe occurred over different years and is related with Columbus's 1^st^ voyage and Pizzaro’s voyage during 15^th^ and 16^th^ centuries. The Mesoamerican gene pool arrived first in Europe in 1506, followed by the Andean gene pool in 1528 [19–20]. Further spreading of this crop to other European countries was very complex with various introductions from different American regions and combined exchange involving Mediterranean and European countries [19]. Currently, common bean is grown worldwide for its edible dry seeds or unripe fruit, either as individual gene pools, or as hybrid forms between the two gene pools [21].

Turkey is considered as one of the world’s biodiversity hotspots and the center of origin for many crops [22–23]. Asian traders were responsible for the introduction of common bean into Turkey from Europe. Since then Turkey hosts hundreds of local landraces of common bean in different geographical provinces [24]. Common bean has now secured a unique place in Turkish agricultural and culinary systems. The annual common bean production was 215,000 tons in 2014 [25], which highlights its importance in Turkish economy and diet, with the Turkish northeast Anatolian region contributing the major part of the production.

One of the world’s big concerns in the twenty-first century, is the possibility to produce enough food for current and future generations while confronting climate change, and adverse environmental factors [26] associated with biotic and abiotic stresses. In order to mitigate these problems, there is a need to identify novel source of useful genetic variability. The investigation of genetic diversity is one of the means to get there as this discipline represents an important tool for assessing populations [22–27] that can be harnessed through breeding and in the process of cultivar development. Common bean landraces are naturally adapted to local environments, they are inherently heterogeneous, and can provide sufficient genetic diversity to sustain crop improvement endeavors [28].

Various genetic diversity studies have been conducted in Turkish common bean landrace germplasm, but they suffered poor sampling of this species’ genome. These studies provided fragmented information showing important weaknesses including: the use of small number of accessions, low number of sampled geographical locations, or a small number of markers which did not cover the whole genome. For instance, Khaidizar et al. [29] studied 38 Turkish common bean landraces and reported a mean genetic similarity of 0.585 but, this study focused only on a specific part of Turkey i.e. Northeast Anatolian region. Another study reported genetic diversity of 30 genotypes from two districts of Van province i.e., Ercis and Gevas [30], while Nemli et al. [31] used iPBS markers for the characterization of the 67 Turkish common bean landraces, and, in their recent work, Nemli et al. [32] used SNP markers for the determination of diversity in the Turkish common bean.

The objectives of this study were therefore, to comprehensively investigate the level of genetic diversity and population structure of Turkish common bean germplasm using a larger germplasm with a greater number of high throughput whole-genome markers relative to previous works. To achieve these goals, we used a high number of SilicoDArT markers detected by DArTseq approach, and phenotypic data information in a mini-core collection of Turkish common bean landrace accessions collected from 19 different geographical regions throughout the Turkish territory.

## 2. Material and methods

### 2.1. Plant material

A diversity panel was assembled consisting of natural populations of 177 common bean landraces and 6 commercial cultivars with 5 scarlet runner bean landraces collected by a group of researchers (Baloch FS and Çiftçi V) from various farmers’ fields in different geographical provinces across Turkey. The sampling sites covered a wide range of natural eco-geographical locations (Table 1) under different latitudes and variable ecological conditions i.e. soil type, rainfall, temperature, and water availability. A mini core collection of common bean population was established and grown at the Abant izzet Baysal University, Turkey. A single plant was selected from each accession, and the selections grown under field conditions in augmented design for two consecutive years during 2014 and 2015, applying single plant selection and selfing. To increase seeds for further trials and genotyping purposes, all single plants selected were grown in year 2016 in 2m long single rows 50cm apart, with 10cm between plants within a row. In all trials, local standard agronomic practices were applied, and the commercial cultivars has been used as control group in earlier studies [29] and they are developed from landraces through single plant selection and represent different regions of Turkey. Growth habit and seed weight of each accession were determined as suggested by Singh et al. [12], and used for phenotypic characterization. Similarly, various morphological characters like flower color, bracteole size, bracteole shape, pod shape and degree of curvature, leaf shape and leaf color were taken according to IBPGR descriptors for *Phaseolus* [33].

**Table 1:** Passport data of Turkish common bean accessions.

Accession Number	Names of Landraces	Collection Site	District	Village		Altitude (m)	Coordinates
1	Bingol -1	Bingöl	Genç	Selvi Beldesi		964	38° 34319 / 40° 18917
2	Bingol -6	Bingöl	Ilıcalar	Merkez		1161	38° 58893 / 40° 40699
3	Bingol -7	Bingöl	Merkez	Alatepe		1154	39° 03502 / 40° 45401
4	Bingol -11	Bingöl	Merkez	Çobantaşı		1542	39° 04033 / 40° 48557
5	Bingol -16	Bingöl	Adaklı	Gökçeli		1335	39° 12738 / 40° 25142
6	Bingol -18	Bingöl	Kiğı	Güneyağıl		1489	39^0^ 17427 / 40^0^ 20136
7	Bingol -25	Bingöl	Solhan	Kavaklıdere		1176	38^0^ 55287 / 40^0^ 56822
8	Bingol -33	Bingöl	Yedisu	Şen Mezrası		-	-
9	Bingol -36	Bingöl	Yedisu	Muz		-	-
10	Bingol -44	Bingöl	Yedisu	Kürdan		-	-
11	Bingol -45	Bingöl	Yedisu	Kürdan		-	-
12	Bingol -52	Bingöl	Yedisu	Eski Balta		-	-
13	Bingol -53	Bingöl	Yedisu	Eski Balta		-	-
14	Bingol -58	Bingöl	Yedisu	Kara Polat		-	-
15	Bingol -60	Bingöl	Yedisu	Döşengi		-	-
16	Bingol -61	Bingöl	Yedisu	Kara Polat		-	-
17	Bingol -63	Bingöl	Yedisu	Güzgülü		-	-
18	Bingol -65	Bingöl	Karlıova	Üçevler		-	-
19	Hakkari-7	Hakkâri	Merkez	Otluca		2054	37° 36246 / 43° 42370
20	Hakkari -11	Hakkâri	Merkez	Üzümcü		2097	37° 36332 / 43° 42526
21	Hakkari -12	Hakkâri	Merkez	Üzümcü		2097	37° 36332 / 43° 42526
22	Hakkari -13	Hakkâri	Merkez	Ağaçdibi		2097	37° 29370 / 43° 38184
23	Hakkari -16	Hakkâri	Merkez	Çimenli		1137	37° 29096 / 43° 37693
24	Hakkari -20	Hakkâri	Merkez	Üzümcü		1135	37^0^ 29773 / 43^0^ 34389
25	Hakkari -23	Hakkâri	Merkez	Taşbaşı		970	37° 23929 / 43° 29723
26	Hakkari -28	Hakkâri	Çukurca	Narlı		875	37° 16013 / 43° 35195
27	Hakkari -31	Hakkâri	Merkez	Bay		1832	37^0^ 32687 / 43^0^ 43333
28	Hakkari -37	Hakkâri	Merkez	Merzan		1993	37° 34095 / 43° 42308
29	Hakkari -38	Hakkâri	Merkez	Merzan		1993	37^0^ 34095 / 43^0^ 42308
30	Hakkari -39	Hakkâri	Merkez	Merzan		1993	37^0^ 34095 / 43^0^ 42308
31	Hakkari -43	Hakkâri	Merkez	Durankaya		1764	37° 33418 / 43° 37329
32	Hakkari -44	Hakkâri	Merkez	Durankaya		1764	37° 33418 / 43° 37329
33	Hakkari -51	Hakkâri	Merkez	Rezan		1601	37^0^ 42104 / 43^0^ 56276
34	Hakkari -55	Hakkâri	Yüksekova	Bağışlı		1811	37^0^ 43279 / 44^0^ 02206
35	Hakkari -59	Hakkâri	Yüksekova	Armutdüzü		2090	37° 40771 / 43° 57535
36	Hakkari -63	Hakkâri	Yüksekova	Su Üstü		1955	37° 35208 / 43° 04488
37	Hakkari -65	Hakkâri	Yüksekova	Büyük Çiftlik		1955	37° 35208 / 43° 04488
38	Hakkari -69	Hakkâri	Yüksekova	Merkez		1915	37° 32928 / 44° 08427
39	Hakkari -71	Hakkâri	Şemdinli	Güzelkonak		1724	37° 25223 / 44° 29056
40	Hakkari -76	Hakkâri	Merkez	Üzümcü		1135	37° 29773 / 43° 34389
41	Tokat-83	Tokat	-	-		-	-
42	Maras-92	K.maras	-	-		-	-
43	Bitlis-5	Bitlis	Hizan	Merkez		1629	38^0^ 13424 / 42^0^ 21614
44	Bitlis-14	Bitlis	Hizan	Akbıyık		1522	38° 11967 / 42° 20644
45	Bitlis-16	Bitlis	Hizan	Yemişli		1638	38° 12806 / 42° 21679
46	Bitlis-22	Bitlis	Hizan	Bahçelievler		1521	38° 12806 / 42° 21679
47	Bitlis-25	Bitlis	Hizan	Kalkanlı		2004	38° 07704 / 42° 37670
48	Bitlis-35	Bitlis	Hizan	Soğuksu		1365	38° 06783 / 42° 33292
49	Bitlis-40	Bitlis	Hizan	Gayda		1271	38° 10051 / 42° 22985
50	Bitlis-46	Bitlis	Tatvan	Yolalan		1645	38° 16080 / 42° 18559
51	Bitlis-48	Bitlis	Merkez	Çınarbaşı		1710	38° 15861 / 42° 17972
52	Bitlis-53	Bitlis	Merkez	Kuşlu		1615	38° 19739 / 42° 14841
53	Bitlis-66	Bitlis	Mutki	Yumrumeşe		1459	38° 26765 / 41° 51660
54	Bitlis-69	Bitlis	Mutki	Kavakbaşı		1303	38° 28884 / 41° 48924
55	Bitlis-76	Bitlis	Mutki	Çiftlikyol		1259	38° 30098 / 41° 46302
56	Bitlis-79	Bitlis	Mutki	Eller		1423	38° 28878 / 41° 43845
57	Bitlis-81	Bitlis	Güroymak	Yazlıkonak		1810	38° 30257 / 42° 07150
58	Bitlis-90	Bitlis	Güroymak	Aşağıkolbaşı		1655	38° 32695 / 42° 06804
59	Bitlis-94	Bitlis	Güroymak	Arpacık		1700	38° 30930 / 42° 05787
60	Bitlis-97	Bitlis	Güroymak	Kuştaşı		2002	38° 29645 / 42° 04575
61	Bitlis-103	Bitlis	Tatvan	Taşdemir		1828	38° 27451 / 42° 23777
62	Bitlis-105	Bitlis	Tatvan	Çamaltı		1728	38° 27483 / 42° 26602
63	Bitlis-111	Bitlis	Tatvan	Reşadiye		1689	38° 29404 / 42° 32232
64	Bitlis-114	Bitlis	Merkez	Çınarbaşı		1459	38° 26765 / 42° 51660
65	Bitlis-115	Bitlis	Mutki	Yumrumeşe		2002	38° 29645 / 42° 04575
66	Bitlis-117	Bitlis	Merkez	Kuşlu		1615	38° 19739 / 42° 14841
67	Bitlis-118	Bitlis	Tatvan	Kırkbulak		1752	38° 24726 / 42° 16166
68	Bitlis-119	Bitlis	Hizan	Yemişli		1638	38° 12806 / 42° 21679
69	Bitlis-120	Bitlis	Merkez	Yolalan		1543	38° 17889 / 42° 15891
70	Bitlis-121	Bitlis	Mutki	Yumrumeşe		1459	38° 26765 / 41° 51660
71	Bitlis-124	Bitlis	Güroymak	Yazlıkonak		1615	38° 19739 / 42° 14841
72	Malatya -3	Malatya	Doğanşehir	Erkenek Bel.		1388	37° 55785 / 37° 56501
73	Malatya-13	Malatya	Doğanşehir	Kurucaova Bel		1369	37° 59707 / 38° 01503
74	Malatya -14	Malatya	Doğanşehir	Savaklı		1364	38^0^ 02576 / 37^0^ 54593
75	Malatya -18	Malatya	Doğanşehir	Elmalı		1410	38^0^ 03339 / 37^0^ 44688
76	Malatya -25	Malatya	Doğanşehir	Çığlık		1235	38^0^ 06477 / 37^0^ 55440
77	Malatya -28	Malatya	Doğanşehir	Güroba		1459	38^0^ 05052 / 37^0^ 57494
78	Malatya -32	Malatya	Doğanşehir	Çömlekoba		1370	38^0^ 05372 / 37^0^ 56691
79	Malatya -33	Malatya	Doğanşehir	Polat Bel.		1270	38^0^ 09447 / 37^0^ 51215
80	Malatya -45	Malatya	Akçadağ	Ören		1158	38^0^ 14905 / 37^0^ 55605
81	Malatya -50	Malatya	Hekimhan	Çayevleri Mah.		1457	38^0^ 48854 / 37^0^ 54964
82	Malatya -51	Malatya	Yeşilyurt	Aşağıköy		1456	38^0^ 09010 / 38^0^ 18332
83	Malatya -52	Malatya	Doğanşehir	Merkez		1280	38^0^ 06477 / 37^0^ 55440
84	Malatya -59	Malatya	Doğanşehir	Kurucaova		1369	37^0^ 59707 / 38^0^ 01503
85	Malatya -71	Malatya	Doğanşehir	Güroba		1465	38^0^ 05052 / 37^0^ 57494
86	Tunceli-1	Tunceli	Mazgirt	Merkez		1122	39° 00014 / 39° 34766
87	Tunceli -5	Tunceli	Ovacık	Yeşilova		1289	39° 20037 / 39° 05286
88	Tunceli -11	Tunceli	Pertek	Beydamı		-	-
89	Van-1	Van	Gürpınar	Merkez		1748	38° 19126 / 43° 22555
90	Van-11	Van	Çatak	Elmacı		1807	38° 04867 / 43° 04475
91	Van-13	Van	Çatak	Bilgi		1702	38° 05736 / 43° 15575
92	Van-17	Van	Çatak	Bilgi		1702	38° 05736 / 43° 15575
93	Van-19	Van	Çatak	Alacayar		1629	38° 01890 / 43° 08884
94	Van-25	Van	Çatak	Merkez		1502	38° 00451 / 43° 03619
95	Van-27	Van	Çatak	Merkez		1783	38° 00721 / 43° 04473
96	Van-29	Van	Başkale	Albayrak		2072	38° 08452 / 44° 12332
97	Van-33	Van	Başkale	Çaldıran		2005	37° 47409 / 44° 07448
98	Van-36	Van	Başkale	Belliyurt		1876	37° 49064 / 44° 06905
99	Van-42	Van	Erciş	Merkez		1704	39° 01746 / 43° 21668
100	Van-47	Van	Erciş	Merkez		1689	39° 00036 / 43° 21362
101	Van-51	Van	Başkale	Barış		2244	38° 01147 / 43° 39146
102	Van-64	Van	Bahçesaray	Ünlüce		1702	38° 31128 / 42° 19587
103	Van-65	Van	Bahçesaray	Ünlüce		1702	38° 31128 / 42° 19587
104	Van-68	Van	Bahçesaray	Elmayaka		1705	38° 30546 / 42° 19126
105	Van-59	Van	Çatak	Elmacı		1807	38° 04867 / 43° 04475
106	Elazig-2	Elazığ	Palu	Seydilli		877	38° 41578 / 39° 53162
107	Elazig -7	Elazığ	Palu	Gömeçbağlar		956	38° 37887 / 39° 51625
108	Elazig -9	Elazığ	Palu	Keklikdere		870	38° 36885 / 39° 49865
109	Elazig -10	Elazığ	Palu	Baltaşı		919	38° 35361 / 39° 47344
110	Elazig -14	Elazığ	Maden	Gezin		919	38° 35361 / 39° 47344
111	Elazig -16	Elazığ	Maden	Kızıltepe		1291	38° 28865 / 39° 31155
112	Elazig -25	Elazığ	Maden	Yıldızhan		1313	38° 21174 / 39° 22660
113	Elazig -27	Elazığ	Sivrice	Başkaynak		1390	38° 22855 / 39° 22217
114	Elazig -29	Elazığ	Sivrice	Elmasuyu		1364	38° 24728 / 39° 23341
115	Elazig -30	Elazığ	Maden	Gezin		1350	38° 30760 / 39° 33182
116	Elazig -34	Elazığ	Maden	Yeşilova		1503	38° 32905 / 39° 33695
117	Elazig -36	Elazığ	Maden	Küçükova		1410	38° 32551 / 39° 32526
118	Elazig -39	Elazığ	Maden	Gezin		1350	38° 30760 / 39° 33182
119	Mus-1	Muş	Malazgirt	Gülkuru		1607	39° 05869 / 42° 38738
120	Mus-2	Muş	Bulanık	Güllüova		1550	39° 03619 / 42° 19105
121	Mus-7	Muş	Bulanık	Güllüova		1550	39° 03619 / 42° 19105
122	Mus-10	Muş	Bulanık	Balotu		1489	39° 06752 / 42° 08046
123	Mus-11	Muş	Bulanık	Balotu		1489	39° 06752 / 42° 08046
124	Mus-15	Muş	Bulanık	Değirmensuyu		1514	39° 10268 / 42° 05099
125	Mus-18	Muş	Korkut	Sazlıkbaşı		1293	39° 40424 / 41° 58975
126	Mus-22	Muş	Hasköy	Merkez		1315	38° 38175 / 41° 46056
127	Mus-27	Muş	Hasköy	Azıklı		1369	38° 38595 / 41° 44016
128	Mus-28	Muş	Hasköy	Kültür		1278	38° 40889 / 41° 41773
129	Mus-34	Muş	Merkez	Akpınar		1400	39° 10591 / 41° 30486
130	Mus-39	Muş	Varto	Tepeköy		1280	39° 05383 / 41° 30168
131	Mus-41	Muş	Varto	Tepeköy		1280	39° 05383 / 41° 30168
132	Mus-42	Muş	Varto	Özenç		1468	39° 06895 / 41° 30281
133	Mus-43	Muş	Varto	Taşçı		1577	39° 12636 / 41° 23917
134	Mus-46	Muş	Bulanık	Güllüova		1550	39° 03619 / 42° 19105
135	Mus-48	Muş	Bulanık	Güllüova		1550	39° 03619 / 42° 19105
136	Mus-49	Muş	Bulanık	Güllüova		1550	39° 03619 / 42° 19105
137	Mus-50	Muş	Bulanık	Balotu		1489	39° 06752 / 42° 08046
138	Mus-51	Muş	Bulanık	Adıvar		1463	38° 13447 / 42° 10513
139	Mus-52	Muş	Hasköy	Merkez		1350	38° 13447 / 42° 10513
140	Mus-53	Muş	Hasköy	Azıklı		1369	38° 38595 / 41° 44016
141	Sivas-3	Sivas	Suşehri	Arpacı		1050	40° 957 / 38° 539
142	Sivas-4	Sivas	Suşehri	Günlüce		1050	40° 957 / 38° 539
143	Sivas-7	Sivas	Suşehri	Akşar		1050	40° 957 / 38° 539
144	Sivas-12	Sivas	Hafik	Yakaboyu		1350	39° 510 / 37° 230
145	Sivas-13	Sivas	Kangal	Akpınar		1540	39° 130 / 37° 240
146	Sivas-16	Sivas	Divriği	Arıkbaşı		1250	39° 240 / 38° 70
147	Sivas-17	Sivas	İmranlı	Başlıca		1650	39° 5248 / 38° 758
148	Sivas-18	Sivas	İmranlı	Gökdere		1650	39° 5248 / 38° 758
149	Sivas44	-	-	-		-	-
150	Sivas62	-	-	-		-	-
151	Sivas69	Sivas	-	-		-	-
152	Sivas-70	Sivas	-	-		-	-
153	Bilecik-1	Bilecik	Pazaryeri	Dereköy		786	39° 59’ 38” / 29° 54’ 41”
154	Bilecik-2	Bilecik	Pazaryeri	Günyurdu		805	40° 0’ 5.9” / 29° 54’ 9”
155	Bilecik-6	Bilecik	Pazaryeri	Dereköy		876	39° 59’ 38’ / 29° 54’ 41”
156	Bilecik-7	Bilecik	Pazaryeri	Dereköy		876	39° 59’ 38’ / 29° 54’ 41”
157	Bilecik-8	Bilecik	Pazaryeri	Dereköy		876	39° 59’ 38’ / 29° 54’ 41”
158	Bilecik-10	Bilecik	Pazaryeri	Dereköy		876	39° 59’ 38’ / 29° 54’ 41”
159	Balikesir-3	Balıkesir	Manyas	Salur Mah.		29	40°05’51” / 27°56’11”
160	Balikesir -4	Balıkesir	Manyas	Akçaova Mah.		30	40°07’16” / 27°51’18”
161	Balikesir -5	Balıkesir	İvrindi	Ayaklı Köyü		404	39.516°/ 27.364°
162	Balikesir -6	Balıkesir	İvrindi	Ayaklı Köyü		403	39.516°/ 27.364°
163	Balikesir -17	Balıkesir	Sındırgı	Kürendere		1051	39.313° / 28.571°
164	Balikesir -18	Balıkesir	Sındırgı	Kürendere		1051	39.313° / 28.571°
165	Balikesir -19	Balıkesir	Sındırgı	Kürendere		1051	39.313° / 28.571°
166	Balikesir -20	Balıkesir	Sındırgı	Kürendere		1051	39.313° / 28.571°
167	Duzce -1	Düzce	Merkez	Derdin		859	40.711° / 31.228°
168	Duzce -9	Düzce	Merkez	Darıca Mah.		163	40° 49’ 18” / 31°10’26”
169	Yalova-13	Yalova	Çiftlikköy	Kabaklı		125	40°39′30″ / 29°24′36″
170	Yalova-20	Yalova	Çınarcık	Ortaburun		689	40°37′04″ / 29°09′00″
171	Yalova-21	Yalova	Çınarcık	Ortaburun		688	40°37′04″ / 29°09′00″
172	Erzincan-1	Erzincan	Refahiye	Merkez		1589	39° 544 / 38° 467
173	Erzincan -3	Erzincan	Kemah	Gökkaya		1130	39° 3610 / 39° 28
174	Erzincan -4	Erzincan	Kemaliye	Merkez		950	39° 1539 / 38° 2948
175	Erzincan -5	Erzincan	Kemaliye	Akçalı		950	39° 1539 / 38° 2948
176	Bursa-1	Bursa	Yenişehir	Fethiye		335	40.289°/ 29.445°
177	Bursa-22	Bursa	Kestel	Aksu		360	40.169° / 29.317°
178	Dermasyon	Niğde	-	-		-	-
179	Derinkiyu	Niğde	-	-		-	-
180	Civril-Bolu	Bolu	Merkez	Doğancı Mah.		842	40°40′45″ / 31°33′30″
181	Bolu-Goynuik	Bolu	Merkez	Doğancı Mah.		842	40°40′45″ / 31°33′30″
182	Moralaca	Bolu	Merkez	Doğancı Mah.		842	40°40′45″ / 31°33′30″
183	Akman×						
184	Goynük×						
185	Ksracsehir×						
186	Onceler×						
187	Goksun×						
188	Akdag×						

×Commercial cultivars

### 2.2. DNA extraction

Genomic DNA was extracted from 2-week old seedlings derived from the 188 selfed mini core selections, according to modified CTAB protocol of Doyle and Doyle, [34] with some modifications of Baloch et al. [35]. The quality and quantity of extracted genomic DNA was checked by using DS-11 FX series spectrophotometer/fluorometer (Denovix, Wilmington, DE, USA) and further confirmed by agarose gel electrophoresis (i.e. 0.8% agarose gel). DNA extraction was repeated for some samples until high-quality DNA was obtained. High-quality DNA was further diluted to a final concentration of 50 ng μl^-1^. The DNA samples were processed at Diversity Array Technology Pty, Ltd, Australia (http://www.diversityarrays.com/) for DArTseq analyses using genotyping-by-sequencing platform.

### 2.3. DArTseq analysis

DArTseq represents a combination of complexity reduction method and sequencing of resulting representations on next generation sequencing platforms [36–37–38] and facilitates the selection of genome fractions corresponding to various active genes [39] which are associated with various traits of interest in the plants. Optimization of this technology for the common bean was achieved by considering both fractions of selected genome and size of the representation. PstI-MseI was used in this complexity reduced method. Processing of DNA samples was performed in digestion/ligation reactions principally following Kilian et al. [37]. Amplification of mixed fragments (PstI–MseI) was performed in 30 rounds of PCR using following reaction conditions: (I) 94°C for 1 min, (II) 94°C for 20 s, (III) ramp 2.4°C /s to 58°C, (IV) 58°C for 30 s, (V) ramp 2.4°C /s to 72°C, (VI) 72°C for 45 s, (VII) repeat steps 2 to 6 29 times, (VIII) 72°C for 7 min, (IX) hold at 10°C [37]. After PCR, equimolar amounts of this amplified product were taken and bulked from each sample of the 96-well microtiter plate. This amplified and bulked product were then applied to c-Bot (Illumina) bridge PCR followed by sequencing on an Illumina Hiseq2000. A total of 77 cycles were run for the sequencing (single read). Resulted sequences from each lane were processed through the application of proprietary DArT analytical pipelines [39]. In the primary pipeline the fastq files are first processed to filter away poor quality sequences, applying more stringent selection criteria to the barcode region compared to the rest of the sequence. In that way the assignments of the sequences to specific samples carried in the “barcode split” step are very reliable. Around 4,000,000 sequences per barcode/sample were investigated and used in marker calling. Eventually identical sequences were collapsed into ‘‘fastqcall files”. These files are used in the secondary pipeline for DArT PL’s proprietary SNP and SilicoDArT (presence/absence of restriction fragments in representation) marker calling algorithms (DArTsoft14).

### 2.4. Statistical analysis

#### 2.4.1. DArTseq markers analysis

DArTsoft v.7.4.7 (DArT P/L, Canberra, Australia) was used to analyze all the images from DArTseq platform. DArTseq-detected silicoDArT markers are genetically dominant and were scored in a binary fashion, with 1 and 0, respectively, standing for presence and absence of a restriction fragment in the genomic representation of each sample [22–37]. They were screened according to different parameters such as call rate, polymorphism information content (PIC) and reproducibility. We ignored markers with PIC, reproducibility and call rate values lower than 0.1, 1 and 0.9 respectively, for bioinformatics analyses purposes in order to avoid false inferences.

#### 2.4.2. Genetic diversity analyses

Genetic distances among the evaluated common bean materials were calculated from the proportion of shared alleles obtained from silicoDArT markers by using Euclidean genetic distance coefficients. In addition to the above algorithm, a number of other diversity-relevant metrics were computed. Expected heterozygosity (Hs), overall gene diversity (Ht), and inbreeding coefficient (Fis) were computed using hierfstat R package [40] following the algorithms of Goudet et al. [41] and Yang, [42]. Pairwise kinship coefficients were derived from genomic relationship matrix following the first method described in VanRaden, [43].

Genetic structure was assessed using principal coordinate analysis (PCoA), UPGMA, and model-based Bayesian clustering algorithms. The PCoA is an eigen-analysis of a distance or dissimilarity matrix, and was performed under R software environment [40] by running a multidimensional scaling algorithm on silicoDArT-based Euclidean distance matrix. The UPGMA trees were constructed in R [40] implementing the hclust algorithm, with the UPGMA relevant agglomeration method, on the pair-wise silicoDArT-based Euclidean distance matrix among common bean landraces and modern commercial cultivars; the resulting tree was visualized and edited in iTOL (http://itol.embl.de/; [44]). The Bayesian model-based clustering was implemented in the STRUCTURE software (version 2.3.4; Pritchard et al. [45] following the methodology developed by Evanno et al. [46]. The number of clusters (K) ranging from 1 to 8 were determined by using admixture model and shared allele frequencies. Ten independent runs were set for each K value, and for each run, the initial burn-in period was set to 500 with 500,000 MCMC (Markov chain Monte Carlo) iterations with no prior information on the origin of individuals. The true value of K was estimated using both the posterior probability of the data for a given K (Pritchard et al. [45]) and the Evanno et al. [46] method. To determine suitable number of clusters (number of K; number of subpopulations), we plotted the number of clusters against logarithm probability relative to standard deviation (ΔK) as explained by Evanno et al. [46]. For coherence purposes, resulted populations from the UPGMA and PCoA were named and assigned colors based on clusters identified with model based Structure algorithm. Such a high importance was given to Structure because this algorithm showed more robustness in previous works [47–48]. Genetic differentiation and significance levels were assessed by calculating the pair-wise FST (measure of genetic structure) values using hierfstat R package [40] following the algorithms of Goudet et al. [41] and Yang, [42]. Analysis of variance for phenotypic data was performed by fitting appropriate linear model with fixed effects

$$y_{ij}=\mu +g_{i}+e_{ij}$$ (1)

for **i = 1,….,s** clusters, **j = 1,….,n** genotypes within a cluster **i, y_ij_** is the response variable of genotype **j** in the cluster **i** appropriately expressed as best linear unbiaised estimate from the 2-year augmented design described above, **e** is the residual. Mean comparisons were done by performing Tukey's test or bootstrapping with 1000 resamples and Student t-test as appropriate. Computations were executed using R software.

## 3. Results

### 3.1. silicoDArT markers discovery by GBS

**Fig 1:**
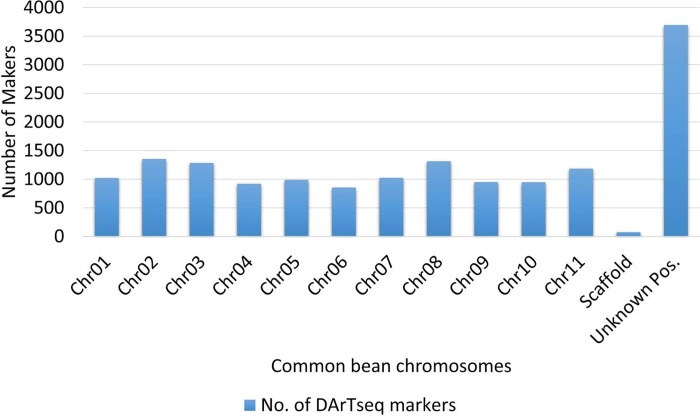
Distributions of silicoDArT markers on different chromosomes of common bean genome.

Whole genome DArTseq profiling of Turkish common bean germplasm was performed, and yielded 15,608 silicoDArT markers (Fig 1) from 3,694 unique sequences. The sequencing company provided the positions of 11,839 markers on the 11 common bean chromosomes according to reference genome. These 11,839 silicoDArT markers were distributed on all chromosomes of common bean (Table 2) with an average of 1,076.27 markers per chromosome; the maximum number of markers (1,354) was found on chromosome 2. The average number of silicoDArT markers/Mbp on all chromosomes was 23.25; chromosome 2 showed the maximum number (27.61), while chromosome 1 had the minimum (19.56). Some silicoDArT markers (3,769) identified in this study have not been previously reported in any linkage studies and therefore they have unknown linkage chromosomal position.

**Table 2:** Distribution of DArTseq markers on different common bean chromosomes.

Chromosome	Chromosome Size (Kbp)^*^	Number of DArTseq Markers	DArTseq marker/Mbp
1	52183.50	1021	19.56
2	49033.70	1354	27.61
3	52218.60	1284	24.58
4	45793.20	920	20.09
5	40237.50	988	24.55
6	31973.20	854	26.70
7	51698.40	1023	19.78
8	59634.60	1314	22.03
9	37399.60	949	25.37
10	43213.20	948	21.93
11	50203.60	1184	23.58
Scaffolds	-	74	-
Total	513589.10	11913	23.25

*Indicates the chromosomal size taken from the reference genome published by Schmutz et al. [5]

**Fig 2:**
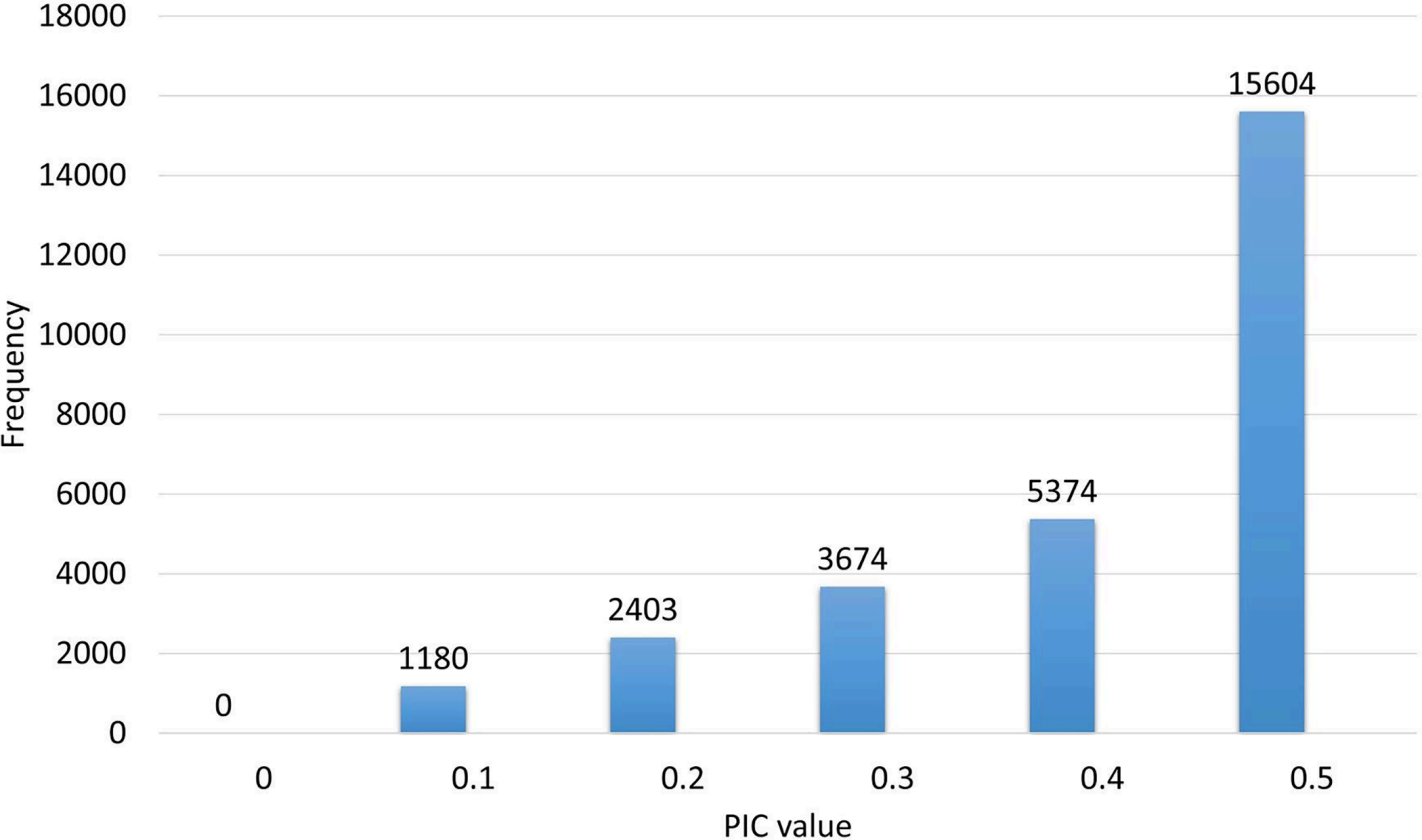
The frequency distribution of polymorphism information contents of silicoDArT markers.

The distribution of PIC values in the original unfiltered silicoDArT marker dataset obtained from the GBS Company is shown in Fig 2. Maximum and minimum PIC values of the whole sample panel were 0.5 and 0.1, respectively, with an average of 0.4. Average call rate was 0.94% with lowest and highest values of 0.76% and 1%, respectively, while the reproducibility was 1 (100% reproducibility). The original silicoDArT marker dataset was filtered to retain 12,557 high quality markers with less than 5% missing data, PIC value greater than 0.10, call rate greater than 0.90, and 100% reproducibility, for use in further analyses in this work.

### 3.2. Genetic diversity and population structure in Turkish common bean

**Fig 3:**
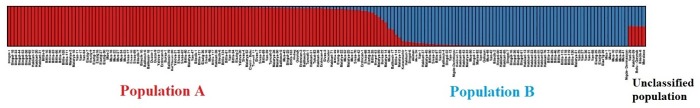
Population structure analysis of Turkish common bean landraces using silicoDArT markers.

**Fig 4:**
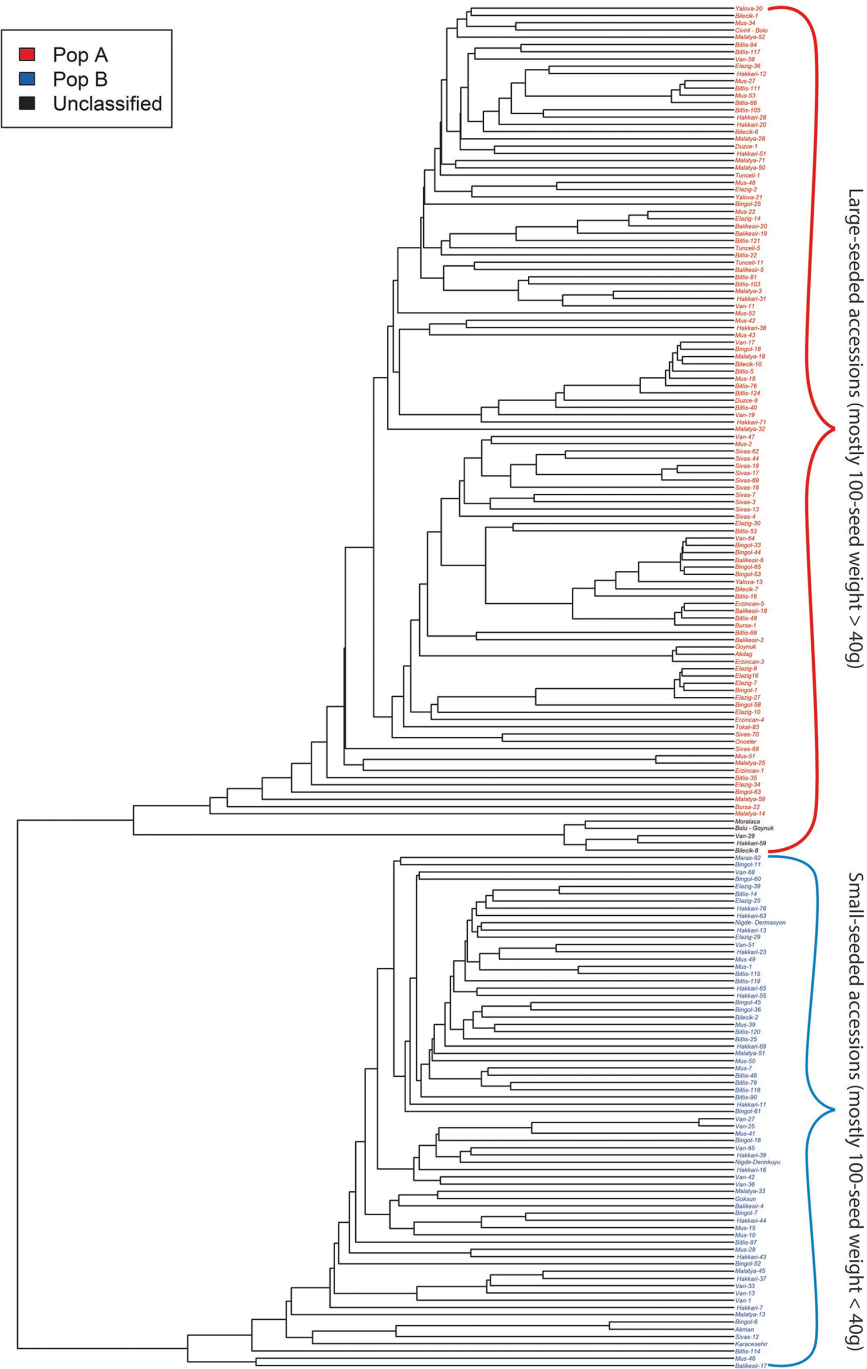
UPGMA based clustering of Turkish common bean accessions based on silicoDArT markers.

**Fig 5:**
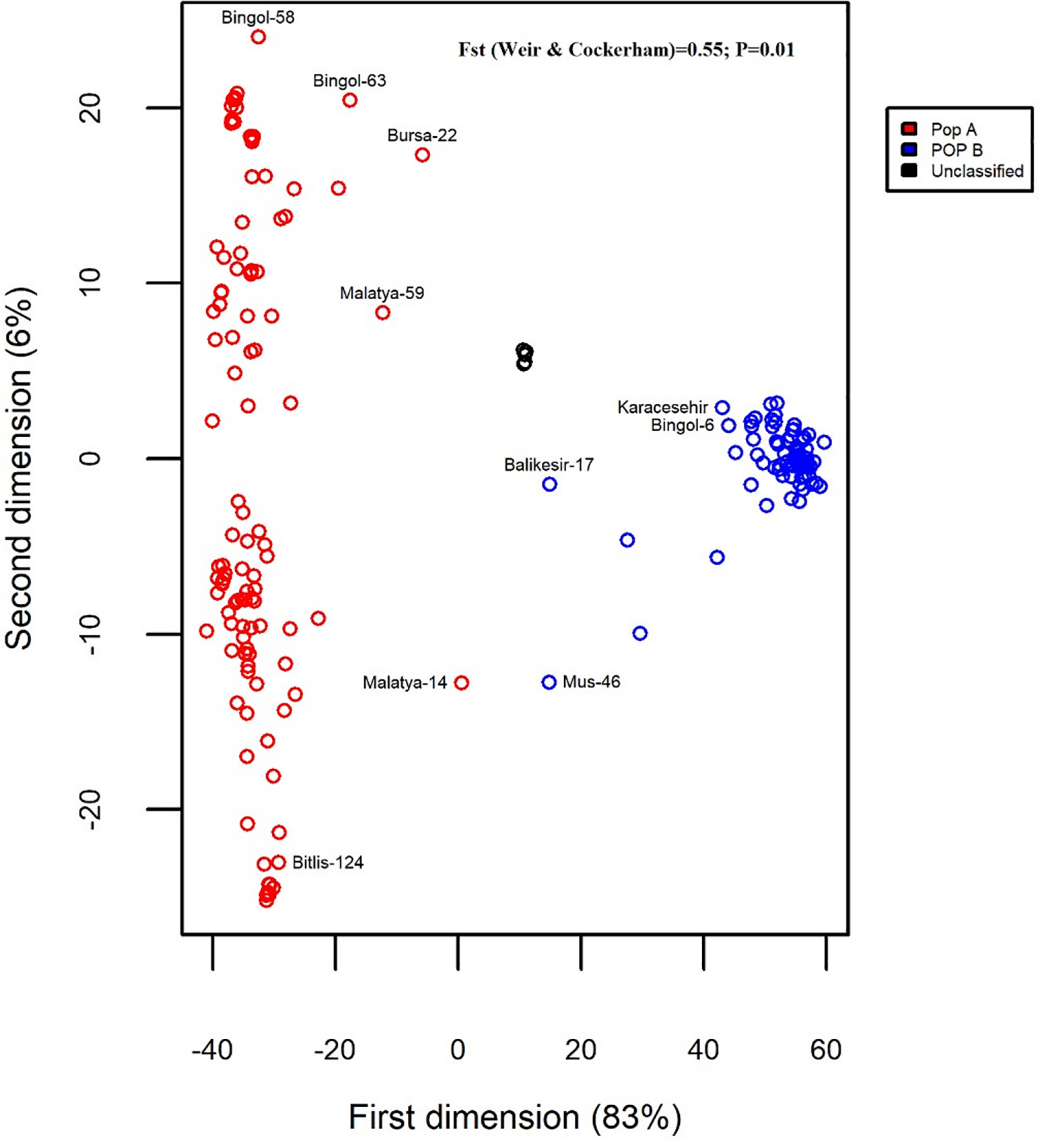
Principal coordinate analysis (PCoA) results based on silicoDArT markers.

The Bayesian clustering model implemented in STRUCTURE software divided the evaluated bean accessions into two main groups: 112 landraces (59.57%) in the group A (red) and 71 landraces (37.76%) in the group B (blue) (Fig 3). Five genotypes (Hakkari-59, Bilecik-8, Bolu-Goynuk, Moralaca, and Van-29) on the right-most end of the graph were successfully discriminated from the rest of the evaluated population, displaying membership coefficients equal to 50% for either population, and were therefore considered as unclassified population (black) as suggested by Habyarimana, [49]. The UPGMA-based tree clearly divided the 188 accessions into the above two main populations A (red) and B (blue) (Fig 4) similar to clustering by model-based structure. Here, also five unclassified landraces were identified and clustered separately. The first two axes of PCoA, explaining 89% of total diversity, divided the landraces into two main populations i.e. A and B (Fig 5), a clustering comparable to the pattern obtained with UPGMA and model-based structure and the unclassified genotypes also made up a separate group.

In order to statistically describe the importance of genetic structure and diversity in the evaluated Turkish common bean germplasm, different diversity metrics such as genetic distance, expected heterozygosity (Hs), overall gene diversity (Ht), pairwise kinship (Φ), Fstatistic (Fst) and inbreeding coefficient (Fis) were computed. For the entire population (188 accessions), the expected heterozygosity was 0.078 and overall gene diversity, Fst and Fis were 0.14, 0.55 and 1, respectively. The expected heterozygosity values for both populations A and B were, respectively, 0.039 and 0.150. (Table 3). The average Euclidean genetic distance for the entire population was 72.59, with a maximum value of 106.35 between Bingol-7 and Hakkari-28 landraces. Average Euclidean genetic distance in population A and population B was 44.62 and 51.40, respectively. Maximum genetic distance within population B was 82.39 between Mus-46 and Bingol-6 landraces, while minimum genetic distance (16.97) within this population was found between landrace Bingol-6 and commercial cultivar Akman. For population A, the maximum genetic distance was 88.09 between landraces Bilecik-8 and Mus-42, while the minimum genetic distance of 7.01 was found between Erzincan-5 and Bitlis-48 landraces. Mean pairwise kinship (Table 4) was higher in population A (-0.00591) than in population B (-0.00967). Kinship coefficients ranged from -0.2233 to 0.8647 in population A and -0.1556 to 0.576 in population B.

**Table 3:** Various diversity metrics for Turkish common bean.

Populations	Hs	Ht	Fst	Fis	Average Euclidean genetic distance	Range of Euclidean genetic distance
Population A	NA	0.039	NA	1	51.40	16.97–82.32
Population B	NA	0.1503	NA	1	44.62	7.01–88.09
Overall population	0.078	0.14	0.55	1	72.59	10.63–106.035

Hs expected heterozygosity, Ht overall gene diversity, Fst: Fstatistic (a measure of genetic structure), Fis: inbreeding coefficient.

**Table 4:** Descriptive statistics for kinship in Turkish common bean germplasm.

Kinship	Min	1st Qu	Median	Mean*	3rd Qu	Max
Pop A	-0.2233	-0.05266	-0.01917	-0.00591 a	0.02055	0.8647
Pop B	-0.1556	-0.05224	-0.02108	-0.00967 b	0.01838	0.576

within the same column, numbers accompanied with different letters are statistically different at the 5% probability level using bootstrapping and t-test.

The analysis of variance showed significant differences between clusters (population A, population B, and unclassified group) in terms of plant height and 100 seed weight (Table 5). Using Tukey test at the 5% probability level, the unclassified population genotypes were comparably tall as population B, both of which were taller than population A. In terms of seed size (100 seed weight), unclassified population (black) landraces displayed biggest seeds (2–3 times bigger than populations A and B) followed by population A, while population B had the smallest seeds. In this study, the 6 known commercial cultivars (Karacesehir, Akman, Goksun, Akdag, Onceler, and Goynuk) were used as control to guide the characterization of the clusters assigned to landraces in terms of growth habit and seed weight. Karacesehir, Akman and Goksun genotypes displayed climber to prostrate growth habit with seed weight less than 40g and Akdag, Onceler, and Goynuk genotypes have indeterminate bushy growth habit with seed weight greater than 40g. Commercial cultivars Karacesehir, Akman, and Goksun clustered with the B group, while Akdag, Onceler, and Goynuk clustered with the A group (Figs 3, 4 and 5), as expected. Within population A, 76% of the accessions displayed 100 seed weight values greater than 40g (ranging from 40 to 68g), while 24% of the accessions had less than 40g (Table 6). Within population B, 75% of the accessions displayed 100 seed weight values lower than 40g, while 25% of the accessions showed values greater than 40g (ranging from 40 to 61g). All the unclassified population landraces showed uniquely big seeds with 100 seed weight ranging from 110.96 to 167.21g. Various morphological characters were also observed to explore the level of diversity more comprehensively and white was the dominant flower color with the small bracteole having intermediate shape bracteole (Table 6). Dominant pod shape of curvature was concave with weak degree of curvature. Mostly terminal leaflet shape was triangular and light green was dominant color in Turkish common bean germplasm.

**Table 5:** Analysis of variance and mean comparison among structure groups.

Traits	PopA	PopB	unclassified	Str MS	Str F	Str P
Plant height	105.02 b	129.00 a	167.62 a	38005.98	15.57	0.0001
100 seed weight	46.23 b	35.29 c	145.09 a	1983.89	4.99	0.03

Str MS, F, P structure mean squares, F and P values

Within the same row, numbers accompanied with same letter are not significantly different using Tukey test at the 5% probability level_._

**Table 6:** Various morphological characteristics in Turkish common bean germplasm.

Genotypes	Flower color	Bracteole Size	Bracteole shape	Pod shape of curvature	Degree of curvature	Leaf shape	Leaf color	*Growth habit	100 seeds weight (g)
Bingol -1	White	Small	Lanceolate	convex	weak	Circular	Medium green	I.B	35.21
Bingol -6	White	Small	intermediate	concave	very slight	quadrangular	Dark green	P	29.53
Bingol -7	White	Medium	Ovate	concave	weak	Circular	Medium green	I.B	37.22
Bingol -11	White	Small	intermediate	convex	very strong	Triangular	Dark green	I.B	34.5
Bingol -16	White	Medium	Lanceolate	convex	medium	Circular	Medium green	C	46.34
Bingol -18	White	Large	Ovate	convex	very slight	quadrangular	Dark green	C	44.11
Bingol -25	White	Small	Ovate	concave	medium	Triangular	Dark green	C	39.48
Bingol -33	White	Medium	Ovate	concave	weak	Triangular	Pale green	I.B	58.32
Bingol -36	White	Medium	intermediate	concave	medium	Circular	Medium green	C	41.38
Bingol -44	White	Small	intermediate	concave	weak	Triangular	Medium green	I.B	56.47
Bingol -45	White	Large	Lanceolate	concave	strong	Circular	Medium green	C	37.58
Bingol -52	White	Small	Lanceolate	convex	medium	Triangular	Pale green	C	26.84
Bingol -53	White	Medium	Ovate	concave	very slight	Triangular	Pale green	I.B	60.74
Bingol -58	White	Small	Ovate	convex	weak	Triangular	Pale green	I.B	39.11
Bingol -60	White	Medium	intermediate	concave	medium	quadrangular	Pale green	C	36.16
Bingol -61	White	Medium	intermediate	concave	weak	Circular	Medium green	C	28.06
Bingol -63	White	Large	intermediate	concave	strong	Circular	Dark green	I.B	47.88
Bingol -65	White	Small	Lanceolate	concave	very slight	Triangular	Pale green	I.B	62.08
Hakkari-7	Purple	Medium	intermediate	concave	strong	Circular	Dark green	C	45.71
Hakkari -11	White	Large	Lanceolate	concave	very strong	Circular	Dark green	C	28.67
Hakkari -12	lilac	Small	Ovate	s-shaped	weak	Triangular	Dark green	C	39.26
Hakkari -13	White	Small	Ovate	concave	strong	quadrangular	Dark green	C	38.08
Hakkari -16	White	Small	intermediate	s-shaped	weak	Circular	Medium green	C	25.54
Hakkari -20	Purple	Small	intermediate	convex	weak	Circular	Dark green	C	39.52
Hakkari -23	Purple	Medium	Ovate	convex	medium	Circular	Dark green	C	21.79
Hakkari -28	White	Small	Ovate	concave	strong	quadrangular	Dark green	C	46.04
Hakkari -31	White	Medium	Ovate	concave	very slight	Triangular	Dark green	C	40.45
Hakkari -37	White	Large	Lanceolate	s-shaped	strong	quadrangular	Dark green	C	35.59
Hakkari -38	White	Large	intermediate	concave	very strong	Triangular	Dark green	C	38.34
Hakkari -39	White	Small	intermediate	convex	strong	Circular	Medium green	C	52.6
Hakkari -43	White	Small	intermediate	convex	very slight	Triangular	Pale green	C	49.6
Hakkari -44	White	Small	Ovate	convex	weak	Triangular	Medium green	P	46.73
Hakkari -51	White	Large	Ovate	concave	weak	Triangular	Dark green	C	32.81
Hakkari -55	White	Small	intermediate	convex	strong	quadrangular	Dark green	C	29.01
Hakkari -59	White	Large	Ovate	concave	strong	quadrangular	Dark green	C	167.21
Hakkari -63	Purple	Small	Ovate	concave	strong	Circular	Medium green	C	18
Hakkari -65	White	Medium	intermediate	concave	strong	Circular	Pale green	C	29.1
Hakkari -69	White	Medium	Lanceolate	s-shaped	weak	quadrangular	Medium green	C	28
Hakkari -71	White	Large	intermediate	convex	medium	Circular	Dark green	C	43.25
Hakkari -76	White	Small	Ovate	concave	medium	Triangular	Pale green	C	29.1
Tokat-83	White	Medium	intermediate	convex	weak	Circular	Pale green	P	52.58
Maras-92	White	Small	Ovate	convex	very slight	Triangular	Pale green	I.B	33.87
Bitlis-5	White	Medium	Ovate	concave	weak	Circular	Medium green	C	44.93
Bitlis-14	Purple	Large	intermediate	concave	strong	Circular	Dark green	C	31.62
Bitlis-16	White	Medium	intermediate	concave	very slight	quadrangular	Medium green	I.B	47.17
Bitlis-22	lilac	Small	intermediate	concave	very slight	Triangular	Medium green	C	41.11
Bitlis-25	White	Medium	Lanceolate	concave	very strong	Circular	Medium green	C	30.48
Bitlis-35	White	Small	Ovate	concave	weak	Triangular	Pale green	C	21.69
Bitlis-40	Purple	Medium	Lanceolate	concave	medium	quadrangular	Dark green	C	49.77
Bitlis-46	White	Medium	Lanceolate	convex	weak	Triangular	Pale green	C	39
Bitlis-48	White	Small	Ovate	concave	weak	Triangular	Pale green	I.B	53.19
Bitlis-53	White	Medium	Lanceolate	concave	weak	quadrangular	Medium green	I.B	59.88
Bitlis-66	Light purple	Small	intermediate	convex	medium	quadrangular	Medium green	C	39.92
Bitlis-69	Light purple	Small	Lanceolate	concave	weak	quadrangular	Pale green	I.B	48.94
Bitlis-76	White	Medium	Lanceolate	concave	weak	Triangular	Dark green	C	51.3
Bitlis-79	White	Medium	intermediate	concave	medium	Circular	Medium green	C	33.31
Bitlis-81	White	Small	Ovate	concave	strong	Circular	Dark green	C	50.55
Bitlis-90	White	Medium	Lanceolate	concave	strong	Circular	Medium green	C	21.6
Bitlis-94	Purple	Large	Lanceolate	concave	very strong	Circular	Pale green	C	42.44
Bitlis-97	White	Medium	Ovate	convex	medium	Circular	Dark green	C	41.82
Bitlis-103	White	Large	intermediate	concave	strong	Circular	Dark green	C	35.27
Bitlis-105	Purple	Medium	intermediate	convex	medium	quadrangular	Medium green	C	36.16
Bitlis-111	lilac	Medium	Ovate	convex	very slight	Triangular	Medium green	C	44.11
Bitlis-114	White	Small	intermediate	convex	medium	Triangular	Dark green	C	33.3
Bitlis-115	White	Small	intermediate	convex	medium	quadrangular	Medium green	C	31.39
Bitlis-117	Purple	Large	Ovate	convex	medium	Triangular	Pale green	C	37.03
Bitlis-118	White	Small	Ovate	convex	very strong	Circular	Pale green	C	37.94
Bitlis-119	White	Small	Ovate	concave	strong	quadrangular	Dark green	C	29.64
Bitlis-120	White	Small	Ovate	concave	medium	Circular	Medium green	I.B	20.13
Bitlis-121	White	Large	Lanceolate	concave	very strong	Circular	Dark green	C	50.82
Bitlis-124	White	Small	Lanceolate	concave	weak	Circular	Medium green	C	40.98
Malatya -3	Purple	Small	Ovate	concave	strong	quadrangular	Medium green	C	34.7
Malatya-13	Purple	Small	Ovate	convex	medium	Triangular	Dark green	C	16.45
Malatya -14	Purple	Medium	Lanceolate	convex	very strong	Circular	Dark green	C	28.5
Malatya -18	White	Small	Lanceolate	concave	weak	Circular	Pale green	C	43.98
Malatya -25	Purple	Medium	intermediate	convex	very strong	Triangular	Pale green	I.B	29.93
Malatya -28	White	Small	Ovate	concave	medium	Triangular	Pale green	C	47.9
Malatya -32	White	Small	Ovate	convex	very slight	Triangular	Pale green	C	43.15
Malatya -33	White	Medium	Lanceolate	concave	weak	quadrangular	Pale green	P	27.44
Malatya -45	White	Medium	intermediate	concave	medium	quadrangular	Pale green	C	38.23
Malatya -50	Purple	Large	Lanceolate	concave	very slight	Circular	Pale green	C	33.19
Malatya -51	White	Small	Lanceolate	concave	strong	Circular	Pale green	C	39.62
Malatya -52	White	Small	intermediate	concave	very slight	quadrangular	Pale green	C	49.5
Malatya -59	White	Small	intermediate	concave	very slight	quadrangular	Pale green	I.B	23.54
Malatya -71	Purple	Medium	intermediate	concave	weak	Triangular	Medium green	C	43.51
Tunceli-1	White	Medium	intermediate	concave	medium	quadrangular	Pale green	C	37.24
Tunceli -5	lilac	Medium	intermediate	concave	medium	Triangular	Medium green	C	57.66
Tunceli -11	Purple	Medium	Ovate	concave	weak	Triangular	Medium green	C	41.31
Van-1	White	Medium	Ovate	concave	strong	Triangular	Pale green	C	28.51
Van-11	Purple	Medium	Ovate	concave	very slight	Triangular	Pale green	C	27.74
Van-13	White	Medium	Lanceolate	concave	strong	Circular	Dark green	C	32.45
Van-17	White	Medium	Lanceolate	concave	medium	Circular	Dark green	C	40.91
Van-19	lilac	Small	intermediate	concave	weak	Triangular	Dark green	C	45.28
Van-25	White	Small	Ovate	concave	very strong	quadrangular	Dark green	C	59.87
Van-27	White	Small	Ovate	concave	strong	quadrangular	Dark green	C	56.97
Van-29	White	Large	intermediate	concave	very strong	Triangular	Dark green	C	110.96
Van-33	White	Small	intermediate	convex	medium	Circular	Pale green	C	33.73
Van-36	White	Small	intermediate	concave	medium	Triangular	Pale green	C	56.34
Van-42	White	Small	Ovate	concave	medium	quadrangular	Pale green	C	53.68
Van-47	White	Small	Ovate	convex	very slight	Circular	Pale green	I.B	48.21
Van-51	Purple	Small	Lanceolate	convex	medium	Triangular	Pale green	P	28
Van-64	White	Medium	intermediate	concave	very strong	Triangular	Medium green	C	52.8
Van-65	White	Small	Lanceolate	concave	very slight	Circular	Pale green	P	60.03
Van-68	White	Large	Ovate	concave	weak	Circular	Medium green	C	60.74
Van-59	White	Small	Lanceolate	concave	weak	Triangular	Pale green	C	41.28
Elazig-2	Purple	Small	intermediate	concave	weak	Triangular	Pale green	C	57.5
Elazig -7	White	Medium	intermediate	concave	weak	Triangular	Pale green	I.B	30.64
Elazig -9	White	Small	intermediate	concave	very slight	Triangular	Pale green	I.B	36.39
Elazig -10	White	Small	Lanceolate	concave	medium	Triangular	Medium green	P	48.63
Elazig -14	White	Small	intermediate	concave	strong	quadrangular	Pale green	C	50.88
Elazig -16	White	Small	intermediate	concave	very slight	Triangular	Pale green	P	45.49
Elazig -25	White	Medium	Lanceolate	concave	weak	Triangular	Medium green	C	31.87
Elazig -27	White	Small	Ovate	concave	medium	Triangular	Medium green	I.B	34.08
Elazig -29	White	Small	intermediate	concave	weak	Triangular	Medium green	P	26.94
Elazig -30	White	Small	Lanceolate	concave	medium	Triangular	Dark green	I.B	55.36
Elazig -34	White	Medium	Lanceolate	concave	medium	Circular	Dark green	I.B	37.13
Elazig -36	Purple	Medium	Lanceolate	concave	medium	quadrangular	Medium green	C	48.03
Elazig -39	White	Small	Lanceolate	concave	weak	Circular	Dark green	C	25.9
Mus-1	White	Small	Lanceolate	concave	medium	quadrangular	Dark green	P	31.2
Mus-2	Purple	Small	Lanceolate	concave	medium	quadrangular	Medium green	C	62.5
Mus-7	White	Small	intermediate	concave	medium	quadrangular	Dark green	C	36.86
Mus-10	White	Small	intermediate	concave	weak	Circular	Dark green	P	40.87
Mus-11	White	Small	Lanceolate	concave	medium	Circular	Dark green	C	37.75
Mus-15	White	Medium	intermediate	concave	medium	Circular	Dark green	C	42.37
Mus-18	lilac	Medium	intermediate	concave	strong	Circular	Dark green	C	57.42
Mus-22	White	Small	intermediate	concave	weak	Triangular	Pale green	C	45.42
Mus-27	White	Medium	Lanceolate	convex	medium	Triangular	Pale green	I.B	31.7
Mus-28	White	Medium	intermediate	concave	medium	quadrangular	Dark green	C	41.8
Mus-34	Purple	Medium	intermediate	concave	strong	Circular	Dark green	P	28.39
Mus-39	White	Small	Lanceolate	concave	medium	quadrangular	Dark green	P	50.5
Mus-41	lilac	Small	Lanceolate	concave	strong	quadrangular	Dark green	C	43.97
Mus-42	White	Small	intermediate	concave	medium	quadrangular	Medium green	C	46.43
Mus-43	Purple	Small	intermediate	concave	medium	Triangular	Pale green	C	30.26
Mus-46	Purple	Medium	Lanceolate	concave	weak	Triangular	Pale green	C	37.86
Mus-48	White	Medium	Lanceolate	concave	strong	Triangular	Pale green	C	28.11
Mus-49	White	Small	intermediate	concave	weak	Circular	Pale green	C	25.62
Mus-50	Purple	Small	Ovate	concave	very strong	Circular	Pale green	P	40.6
Mus-51	Purple	Medium	intermediate	convex	weak	quadrangular	Medium green	C	54.13
Mus-52	Purple	Small	Lanceolate	concave	very slight	Circular	Pale green	C	43.18
Mus-53	White	Medium	intermediate	concave	very slight	quadrangular	Pale green	I.B	59.72
Sivas-3	White	Medium	Lanceolate	concave	very slight	quadrangular	Pale green	I.B	44.15
Sivas-4	Purple	Small	Lanceolate	concave	medium	Triangular	Pale green	I.B	52.68
Sivas-7	White	Medium	intermediate	concave	strong	quadrangular	Dark green	P	36.6
Sivas-12	White	Small	intermediate	concave	medium	Circular	Dark green	P	41.25
Sivas-13	White	Small	intermediate	concave	medium	Triangular	Dark green	I.B	43.61
Sivas-16	Light purple	Medium	Lanceolate	concave	medium	Triangular	Dark green	I.B	57.72
Sivas-17	Light purple	Small	Ovate	concave	strong	Circular	Dark green	P	59.09
Sivas-18	White	Small	Lanceolate	concave	medium	quadrangular	Pale green	I.B	59.81
Sivas44	White	Small	intermediate	concave	weak	quadrangular	Pale green	I.B	67.76
Sivas62	White	Small	intermediate	concave	very slight	Triangular	Pale green	I.B	38.81
Sivas69	Light purple	Medium	Lanceolate	concave	weak	Triangular	Pale green	I.B	57.13
Sivas-70	White	Small	intermediate	concave	very slight	quadrangular	Pale green	I.B	40.33
Bilecik-1	Purple	Small	Lanceolate	concave	very slight	Circular	Pale green	C	35
Bilecik-2	White	Medium	Lanceolate	concave	very strong	Triangular	Dark green	P	28.34
Bilecik-6	Purple	Medium	intermediate	concave	weak	quadrangular	Dark green	C	47.54
Bilecik-7	White	Small	Lanceolate	concave	very slight	Triangular	Pale green	P	57.31
Bilecik-8	White	Large	intermediate	concave	very strong	Circular	Dark green	C	139.42
Bilecik-10	White	Large	Lanceolate	concave	medium	quadrangular	Dark green	C	47.51
Balikesir-3	Purple	Small	Lanceolate	concave	medium	quadrangular	Pale green	P	30.41
Balikesir -4	White	Small	intermediate	concave	medium	quadrangular	Pale green	P	52.45
Balikesir -5	Purple	Small	intermediate	concave	medium	quadrangular	Pale green	C	63.43
Balikesir -6	White	Small	Lanceolate	concave	weak	Triangular	Pale green	P	52.29
Balikesir -17	White	Small	intermediate	concave	weak	Triangular	Pale green	C	31.22
Balikesir -18	White	Medium	intermediate	concave	very slight	quadrangular	Pale green	I.B	55.29
Balikesir -19	Purple	Small	Lanceolate	concave	medium	Triangular	Pale green	C	45.27
Balikesir -20	White	Medium	Ovate	concave	medium	Circular	Pale green	C	58.46
Duzce -1	White	Small	Ovate	concave	strong	quadrangular	Pale green	C	47.85
Duzce -9	Light purple	Small	Lanceolate	concave	medium	Triangular	Dark green	C	54.24
Yalova-13	White	Small	Lanceolate	concave	very slight	quadrangular	Pale green	I.B	61.15
Yalova-20	White	Medium	intermediate	concave	very slight	quadrangular	Pale green	C	43.79
Yalova-21	Purple	Small	Lanceolate	concave	very slight	quadrangular	Pale green	C	46.76
Erzincan-1	White	Small	intermediate	concave	very slight	Triangular	Pale green	I.B	42.08
Erzincan -3	White	Small	intermediate	concave	medium	quadrangular	Pale green	I.B	55.43
Erzincan -4	Purple	Small	intermediate	concave	weak	quadrangular	Dark green	I.B	54.28
Erzincan -5	White	Small	intermediate	concave	very slight	quadrangular	Dark green	I.B	51.42
Bursa-1	White	Small	intermediate	concave	weak	Triangular	Dark green	I.B	52.68
Bursa-22	White	Medium	Lanceolate	concave	weak	Triangular	Pale green	I.B	46.1
Dermasyon	White	Small	Lanceolate	concave	weak	Circular	Medium green	P	41.29
Derinkiyu	White	Small	intermediate	concave	medium	Triangular	Pale green	P	57.93
Civril-Bolu	White	Small	Lanceolate	concave	medium	Circular	Dark green	C	46.23
Bolu-Goynuik	White	Large	intermediate	convex	very strong	Circular	Dark green	C	164.27
Moralaca	Red	Large	intermediate	convex	very strong	quadrangular	Dark green	C	143.61
Akman×	White	Medium	Lanceolate	concave	weak	Triangular	Dark green	I.B	27.97
Goynük×	White	Small	intermediate	concave	very slight	Triangular	Dark green	I.B	48.5
Ksracsehir×	Purple	Small	intermediate	concave	very slight	quadrangular	Pale green	P	20.84
Onceler×	Purple	Small	Lanceolate	concave	very slight	Triangular	Pale green	I.B	41.08
Goksun×	White	Medium	Lanceolate	concave	medium	quadrangular	Dark green	P	29.2
Akdag×	White	Small	intermediate	concave	weak	Triangular	Dark green	I.B	50.39

*Growth Habit (C, climber; P, Prostrate; I.B, Indeterminate bush)

×Commercial cultivars

After the seed weight, growth habit, geographical provinces and flower color actively participated in clustering. Population A contains indeterminate bushes, prostrate and climbering genotypes, while population B grouped landraces having only indeterminate and climber growth habit and mostly genotypes in both populations from different provinces clustered with genotypes having similar growth habit. Geographical provinces also played a role in clustering and genotypes belonging to same provinces generally clustered together in both populations. However, it was also observed that genotypes with same provenance also clustered in different groups. For instance, landraces from Hakkari, Mus and Van provinces displayed the phenotypic characteristics of population A but, some of the landraces from these provinces grouped with B population. On the other hand, Elazig, Bitlis and Balikesir provinces provided landraces reflecting the phenotypic characteristics of B population, but some landraces from these provinces clustered in A population. Flower color clearly differentiated the genotypes by clustering white flower genotypes population A and purple, white and lilac colors were invariably found in population B.

## 4. Discussion

### 4.1. DArTseq-generated silicoDArT markers as a genotyping tool

Investigation of genetic diversity is very important because it can provide insight into sources of novel alleles to be used in breeding programs. The use of molecular markers to assess genetic diversity represents a significant breakthrough [50–51–52]. Various types of molecular markers have been employed in an attempt to assess genetic diversity of common bean [9–19–53]. However, DArTseq-generated marker system emerged as a marker of choice for scientists for its high throughput, possibility of whole genome covering [38–54] and because it can be an alternative genotyping tool for the research laboratories having less financial support. In this work, a greater number (12,557) of highly polymorphic silicoDArT markers were used relative to previous studies [55–56] in order to produce more reliable results. For example, Cichy et al. [55] used 84 DArT and 494 SNP markers for the investigation of QTLs for seed color traits in common bean. Valdisser et al. [56] used the 6286 DArTseq generated SNPs markers for the diversity identification in Brazilian common bean. In another study, Valdisser et al. [57] identified a total of 23,748 RAD-SNPs, of which 3357 were found adequate for common bean genotyping.

The distribution of the silicoDArT markers used in this work was generally homogeneous on each common bean chromosome but, the number of markers per chromosome was variable. Chromosome 2 had 11.43%, while 7.21% of the markers were found on chromosome 6. On average, 1,076.27 (6.89%) markers were identified per chromosome. These results are supported by earlier studies [32–56–58] reporting relatively more silicoDArT markers on common bean chromosome 2 and less number of markers on chromosome 6 [58]. However, our results made strong disagreement with Schroder et al. [59], as they found maximum SNP markers on chromosome 8. This may be due to the use of different marker system.

The identification of 24.14% novel markers with unknown positions represents important findings in this study, particularly for the breeding perspectives. Novel markers can be used in genome wide association studies (GWAS) for the discovery of genetic factors of interest. We are conducting multi-location/year morphological experiments and these newly identified markers will be used for common bean GWAS in upcoming years. A good range of PIC value (0.10–0.5) was found in this study, which is in line with previous works on common beans. Valdisser et al. [56] found PIC values from 0.23 to 0.5 using DArTseq markers, Blair et al. [60] found 0.32 using SNPs, Wani et al. [61] obtained 0.22–0.49 with SSR and Kumar et al. [62] achieved 0.22–0.30 using AFLP markers. Mean PIC value (0.4) obtained in our study was much higher than obtained by the Nemli, et al. [32] in their recent work on the characterization of Turkish common bean germplasm with DArTseq-generated SNP markers. The wider PIC range obtained in this work suggests a greater level of variation deriving probably from the use of larger number of high quality markers in a larger and diverse population.

### 4.2. Genetic structure and diversity in Turkish common bean

Whole-genome silicoDArT molecular markers, growth habit, and 100 seed weight were used to characterize the Turkish common bean germplasm. More importance was given to molecular markers as suggested in Habyarimana, [49], because they provide higher clustering accuracy. The three clustering algorithms, model-based structure, UPGMA, and PCoA were implemented and showed a good level of agreement. Three genetic groups, population A, population B, and a group of unclassified population, were identified and represented heterotic groups from which parental lines can be fetched to conduct crossing blocks in the process of common bean genetic improvement.

Genomic inbreeding and kinship coefficient were computed as part of diversity metrics. Within a population, individual genomic inbreeding represents the probability that two alleles at a randomly chosen locus are identical by state, whereas pairwise kinship measures the relatedness represented by the probability that two alleles, one sampled at random from each individual, are identical by state. Therefore, kinship predicts the future level of inbreeding which represents the repository for future genetic diversity.

The overall gene diversity and the range of Euclidean distance (Table 3) were higher in population B, while the pairwise kinship (Table 4) was higher in population A, confirming the higher level of genetic diversity in the population B which can be used with advantage for common bean genetic improvement. The overall gene diversity over the entire germplasm was lower than in Gioia et al. [19] working on the 256 European and 56 American landraces using chloroplast microsatellite (cpSSRs) and nuclear markers (phaseolin and Pv-shatterproof1). However gene diversity in the population B was higher than obtained by Nemli, et al. [32] using DArTseq generated SNP markers in 173 accessions mainly from Turkey. The results in this study are in good agreement with previous findings [9–63–64] in terms of relative importance of the gene diversity of common bean accessions.

The Fst statistic achieved in this work is much higher than in earlier reports by McClean et al. [65] working on USDA core collectıon. These differences can be attributed to the use of a higher number of markers, genotyping more diverse materials from different locations [66], and the use of different sampling approaches in this work. Overall and in both genetic populations, inbreeding coefficient (Fis) was found 1 in this study, and this was expected in virtue of the self-pollinating reproduction system in common bean.

Euclidean distance is a mean quantitative measure of the genetic divergence and can be calculated between populations, species or individuals at DNA sequence level or allele frequency level [67]. Information about the existence of genetic variations and relationships in common bean landraces is very useful for the selection parents to develop new gene recombinations and a much effective germplasm characterization for diverse agriculture [68]. Maximum genetic distance in Turkish germplasm was 106.035 between Bingol-7 and Hakkari-28 landraces. Bingol-7 belongs to population B having small seed size, bushy growth habit, while Hakkari-28 is a climber in nature and contained large seed size and clustered in population A. These landraces can therefore be good candidate parents for the development of improved common bean varieties.

Landraces Bilecik-8 and Mus-42 showed the highest genetic distance within the population A. As Turkish people like seeds with medium to large size in their diet, these landraces can be candidate parents for the development of common bean variety having favorable traits for the consumer. Similarly, we found Erzincan-5 and Bitlis-48 landraces most genetically similar to each other in population A, as they showed minimum pairwise genetic distance. Within the population B, the maximum genetic distance was found between Mus-46 and Bingol-6 landraces, while Bingol-6 and commercial cultivar Akman showed higher levels of similarity. The genetically distinct landraces identified in this work (Bingol-7, Hakkari-28, Bilecik-8, Mus-42, Mus-46 and Bingol-6) within and between both populations represent a great common bean breeding potential for the scientific community to develop improved cultivars according to farmer and consumer interest.

Structure analysis divided the common bean accessions into 2 main populations: population A was the dominant population with 112 accessions and population B was smaller and contained 71 genotypes. Clustering algorithms were in good agreement with statistical inferences made on phenotypic data (growth habit and seed size). Plant height of population B was higher than in population A (Table 5), because population B contained only genotypes having prostrate and climber growth habit while population A contained landraces with indeterminate bush growth habit landraces besides the other two growth habits. Overall, a higher proportion of climber growth habit (61.70% of total accessions) and the prevalence of large seed size were obtained in this study (Table 6), which is in agreement with Blair et al. [18] reporting higher proportion of climber growth habit in large seeded genotypes, and Angioi et al. [53] and Bitocchi et al. [9] showing the predominance of large seed size accessions in Europe. Commercial cultivars used in this study have been also evaluated by Ceylan et al. [69] and their various phenotypic attributes were also documented by them. Akdag, Onceler and Goynuk cultivars have seed weight above 40g and grouped in population A, while Akman, Goksun and Karacasehir has seed weight below 40g and grouped in population B, and this is in agreement with Ceylan et al. [69].

On the average, landraces clustered in the population A contained seed size greater than 40g/100 seeds and landraces present in the population B contained the seed size less than 40g/100 seeds (Table 6). On the other hand, 76% of accessions in population A had seed size greater than 40g/100 seeds, while 74% of accessions in population B had seed size smaller than 40g/100 seeds. According to Singh et al. [12], genotypes having seed size above 40g belongs to Andean gene pool and those with seed size below 40g are called Mesoamerican gene pool accessions. The occurrence of two gene pools in Turkish bean pool were confirmed in previous studies by the Nemli et al. [32]. Similarly, Nemli et al. [32] along with other scientific groups also came across the prevalence of Andean gene pool in Turkey and in Europe [9–53], which strongly supports the findings in the present work. These results are in line with the previous studies [9–19–70] showing that the European common bean germplasm originated from both gene pools.

In this study, 5 genotypes (Hakkari-59, Bilecik-8, Bolu-Goynuk, Moralaca, and Van-29) did not clustered to any population due to membership coefficient (equal to 0.5) and considered as unclassified genotypes as suggested by Habyarimana, [49] and were present on the right-most end of the structure. All of these genotypes reflected average plant height of 145.09 cm and their 100 seed weight ranged between 110.96 to 167.21g and confirms their uniqueness for both phenotypic and genotypic information. Santalla et al. [8] stated that beans having seed weight more than 100g (100 seed) belongs to scarlet runner bean and mostly scarlet runner bean are climber in nature. On the basis of this information, we found that genotypes in unclassified population has 2–3 times higher seed weight and their plant height is much greater than the both populations of common bean. These genotypes also reflected their uniqueness in structure, UPGMA and PCoA by making their separate population. Therefore, we considered these genotypes as scarlet runner bean genotypes on the basis of information provided by Santalla et al. [8]. Scarlet runner bean has been proven as a good source of variations for the improvement of common bean and these five runner bean accessions can be used for developing new superior common bean cultivars in near future.

Various morphological characteristics were also observed by following the IBPGR descriptors for *Phaseolus* [33], white, lilac and purple colored flowers were present in Turkish common bean. 72.67% genotypes reflected white colored flower, 23.49% were purple colored and 3.82% genotypes contained lilac flowers (Table 6). 56.83% genotypes contained small size bracteole and 43.71% genotypes contained intermediate shaped bracteole. Genotypes present in the population A of structure algorithm, UPGMA and PCoA mainly contained white color flowers with small size bracteole and they contained intermediate shaped bracteole. Genotypes in population B reflected diversity in their flower color and they contains white, purple and lilac color flowers and they contains small to medium size bracteole with lanceolate bracteole shape. 55.73% genotypes contained concave pod shape of curvature and 32.40% contained very slight or no degree of curvature. Genotypes in population A contained concave shaped pods with weak degree of curvature. Population B found diverse by clustering genotypes having concave and convex shaped pods with medium to strong degree of curvature. Triangular, circular and quadrangular were the shapes of leaf and Triangular (39.34%) was the most dominant shape of terminal leaf. Population A contained genotypes having triangular and quadrangular shaped leaf and their leaf color varied from pale green to medium green. Population B was the diverse population by clustering all shapes and color of leaf and mostly genotypes in population B contains medium to dark green leaf color. Among the 5 unclassified genotypes, only Moralaca genotypes contains red color flower and remaining contained white flower with large size and ovate shaped bracteole.

The 24% and 26% of individuals in populations A and B, respectively, showing seed size below 40g/100 seeds (for population A) and above 40g/100 seeds (for population B), can possibly be considered as hybrids. A good proportion of landraces were collected from Van, Bitlis and Hakkari provinces of Turkey and these provinces reflected higher level of hybridization than the other provinces. Turkey is not an origin center for common bean and therefore, the possible reasons for the presence of more hybridization events in these provinces may be their closeness with each other that favored horizontal gene transfer in the direction that most satisfied the interests of the farmer and the taste of the consumer. Presence of hybrids in this study are also confirmed by the recent study of Nemli et al. [32] and Ceylan et al. [69], where they investigated hybrids as separate group. The possible hybridization found in this work was higher than reported earlier by Carović-Stanko et al. [70] using Croatian landraces. There was also a disagreement between this work and the findings in Angioi et al. [53] and Gioia et al. [19] that showed higher level of hybridization in European common bean germplasm containing landraces from nearly all European countries. One of the possible reasons behind the presence of higher hybrids in the above studies on European common bean germplasm can involve the use of different molecular marker systems. Angioi et al. [53] used the chloroplast microsatellites and combined them with two nuclear loci for *Pv-shatterproof1* and Phaseolin type, while Gioia et al. [19] used the nuclear and chloroplast microsatellite markers; whole-genome silicoDArT markers were used in the present work.

The findings in this study showed high genetic diversity with both phenotypic and genotypic information. Genetic relatedness was generally low and heterotic groups were identified that can be used for breeding purposes. Morphological information clearly reflected the existence of variation in Turkish common bean germplasm and supported the genotypic information. Seed weight was the main factor in clustering, while growth habit, geographical provinces, and flower color also played active role in the clustering. Now, there is a need to take initiatives and start executing hybridization programs in common bean in order to develop varieties responding to end user preferences. One of the possible successful breeding objectives can be initiated by developing high-yielding dwarf common bean ideotype that is generally preferred by farmers due to less labor requirement and ease of harvesting. A good number of dwarf accessions were identified in this study. Further dwarf common bean cultivars can be developed through hybridization between distant parents with desired traits. In this study, all genotypes of unclassified population reflected phenotypic and genotypic uniqueness and will be used to start various common bean breeding activities in near future. Endeavors in this direction are underway at the Abant Izzet Baysal University, Bolu, Turkey, where a common bean germplasm mini-core is being increased by collecting more landraces within Turkey and from core countries harboring good common bean diversity such as Mexico and Latin American nations. Currently, we are conducting multiyear/location experiments of this mini-core and markers produced in this study will be used to perform genome wide association studies and marker-aided common bean breeding. In perspective, standard Andean and Mesoamerican checks will be integrated in field trials to confirm the results presented herein. We will use the variety of information collected for the identification of molecular markers linked with yield and yield component traits. The appropriate silicoDArT markers for various traits of interest will be cloned and converted into kompetitive allele specific PCR (KASP). Anyone willing to initiate knowledge based breeding program and interested in Turkish common bean genetic resources based on the information generated in this study can contact us.

## References

[1] Gepts P, Bliss FA. Dissemination pathways of common bean (*Phaseolus vulgaris*, Fabaceae) deduced from phaseolin electrophoretic variability. II. Europe and Africa. Econ Bot. 1988; 42(1): 86–104.

[2] Maras M, Šuštar-Vozlič J, Kainz W, Meglič V. Genetic diversity and dissemination pathways of common bean in central Europe. J Am Soc Hortic Sci. 2013; 138(4): 297–305.

[3] Mamidi S, Rossi M, Annam D, Moghaddam S, Lee R, Papa R, et al Investigation of the domestication of common bean (*Phaseolus vulgaris*) using multilocus sequence data. Funct Plant Biol. 2011; 38(12): 953–967. 10.1071/FP11124 32480954

[4] Petry N, Boy E, Wirth JP, Hurrell RF. The potential of the common bean (*Phaseolus vulgaris*) as a vehicle for iron biofortification. Nutrients. 2015: 7(2): 1144–1173. 10.3390/nu7021144 25679229 PMC4344581

[5] Schmutz J, McClean PE, Mamidi S, Wu GA, Cannon SB, Grimwood J, et al A reference genome for common bean and genome-wide analysis of dual domestications. Nature Genet. 2014: 46(7): 707–713. 10.1038/ng.3008 24908249 PMC7048698

[6] Spataro G, Tiranti B, Arcaleni P, Bellucci E, Attene G, Papa R, et al Genetic diversity and structure of a worldwide collection of *Phaseolus coccineus* L. Theor. Appl. Genet. 2011; 122:1281–91. 10.1007/s00122-011-1530-y 21279322

[7] Salinas AD. Genetic resources of Phaseolus beans In: Gepts P (ed) Variation, taxonomy, domestication, and germplasm potentialities in *Phaseolus coccineus*. Springer, Dordrecht 1988; pp. 441–463

[8] Santalla M, Monteagudo AB, González AM, De Ron AM. Agronomical and quality traits of runner bean germplasm and implications for breeding. Euphytica.2004; 135:205–15.

[9] Bitocchi E, Nanni L, Bellucci E, Rossi M, Giardini A, Zeuli PS, et al Mesoamerican origin of the common bean (*Phaseolus vulgaris* L.) is revealed by sequence data. Proc Natl Acad Sci. 2012; 109(14): E788–796. 10.1073/pnas.1108973109 22393017 PMC3325731

[10] Gross BL, Olsen KM. Genetic perspectives on crop domestication. Trends Plant Sci. 2010; 15(9): 529–537. 10.1016/j.tplants.2010.05.008 20541451 PMC2939243

[11] Pickersgill B, Debouck DG. Domestication patterns in common bean (*Phaseolus vulgaris* L.) and the origin of the Mesoamerican and Andean cultivated races. Theor Appl Genet. 2005; 110(3): 432–444. 10.1007/s00122-004-1842-2 15655667

[12] Singh SP, Gepts P, Debouck DG. Races of common bean (*Phaseolus vulgaris*, Fabaceae). Econ Bot. 1991; 45(3): 379–396.

[13] Singh SP, Gutierrez JA, Molina A, Urrea C, Gepts P. Genetic diversity in cultivated common bean: II. Marker-based analysis of morphological and agronomic traits. Crop Sci. 1991; 31(1): 23–29.

[14] Rodriguez M, Rau D, Angioi SA, Bellucci E, Bitocchi E, Nanni L, et al European *Phaseolus coccineus* L. landraces: population structure and adaptation, as revealed by cpSSRs and phenotypic analyses. PloS one. 2013;8(2): e57337: 10.1371/journal.pone.0057337 23451209 PMC3579852

[15] Kwak M, Gepts P. Structure of genetic diversity in the two major gene pools of common bean (*Phaseolus vulgaris* L., Fabaceae). Theor Appl Genet. 2009; 118(5): 979–992. 10.1007/s00122-008-0955-4 19130029

[16] Rossi M, Bitocchi E, Bellucci E, Nanni L, Rau D, Attene G, et al Linkage disequilibrium and population structure in wild and domesticated populations of *Phaseolus vulgaris* L. Evol Appl. 2009; 2(4): 504–522. 10.1111/j.1752-4571.2009.00082.x 25567895 PMC3352449

[17] Bitocchi E, Rau D, Bellucci E, Rodriguez M, Murgia ML, Gioia T, et al Beans (*Phaseolus* ssp.) as a Model for Understanding Crop Evolution. Front Plant Sci. 2017; 8: 10.3389/fpls.2017.00722 28533789 PMC5420584

[18] Blair MW, Giraldo MC, Buendia HF, Tovar E, Duque MC, Beebe SE. Microsatellite marker diversity in common bean (*Phaseolus vulgaris* L.). Theor Appl Genet. 2006; 113(1): 100–109. 10.1007/s00122-006-0276-4 16614831

[19] Gioia T, Logozzo G, Attene G, Bellucci E, Benedettelli S, Negri V, et al Evidence for introduction bottleneck and extensive inter-gene pool (Mesoamerica x Andes) hybridization in the European common bean (*Phaseolus vulgaris* L.) germplasm. PLoS One. 2013 1;8(10):e75974: 10.1371/journal.pone.0075974 24098412 PMC3788063

[20] Rodiño AP, Santalla M, González AM, De Ron AM, Singh SP. Novel genetic variation in common bean from the Iberian Peninsula. Crop sci. 2006; 46(6): 2540–2546.

[21] Chávez-Servia JL, Heredia-García E, Mayek-Pérez N, Aquino Bolaños EN, Hernández-Delgado S, Carrillo-Rodríguez JC, et al Diversity of Common Bean (*Phaseolus vulgaris* L.) Landraces and the Nutritional Value of their Grains In: Goyal Dr Aakash, editor. Grain Legumes; InTech, 10.5772/63439

[22] Baloch FS, Alsaleh A, Shahid MQ, Çiftçi V, de Miera LE, Aasim M, et al A whole genome DArTseq and SNP analysis for genetic diversity assessment in durum wheat from central fertile crescent. PloS one. 2017;12(1): e0167821: 10.1371/journal.pone.0167821 28099442 PMC5242537

[23] Tan A. Current status of plant genetic resources conservation in Turkey In International Symposium on In Situ Conservation of Plant Genetic Diversity, Antalya (Turkey) 1988 Central Research Institute for Field Crops 5–16.

[24] Şehirali S. Yemeklik Dane Baklagiller Ankara Üniversitesi Ziraat Fakültesi Class Book, no: 1089; 1988.

[25] Food and Agriculture Organization of the United Nations (FAO). (2014). The food and agricultural commodities. [Online]. Available: http://faostat.fao.org/site/339/default.aspx. Accessed [28 May 2017]

[26] Mickelbart MV, Hasegawa PM, Bailey-Serres J. Genetic mechanisms of abiotic stress tolerance that translate to crop yield stability. Nature Rev Genet. 2015; 16(4): 237–251. 10.1038/nrg3901 25752530

[27] Cömertpay G, Baloch FS, Kilian B, Ülger AC, Özkan H. Diversity assessment of Turkish maize landraces based on fluorescent labelled SSR markers. Plant Mol Biol Report. 2012; 30(2): 261–274.

[28] Dwivedi SL, Ceccarelli S, Blair MW, Upadhyaya HD, Are AK, Ortiz R. Landrace germplasm for improving yield and abiotic stress adaptation. Trends Plant Sci. 2016; 21(1): 31–42. 10.1016/j.tplants.2015.10.012 26559599

[29] Khaidizar MI, Haliloglu K, Elkoca E, Aydin M, Kantar F. Genetic diversity of common bean (*Phaseolus vulgaris* L.) landraces grown in northeast Anatolia of Turkey assessed with simple sequence repeat markers. Turk J Field Crops. 2012; 17(2): 145–150.

[30] Sarıkamış G, Yaşar F, Bakır M, Kazan K, Ergül A. Genetic characterization of green bean (*Phaseolus vulgaris*) genotypes from eastern Turkey. Genet Mol Res. 2009; 8(3): 880–887. 10.4238/vol8-3gmr605 19731210

[31] Nemli S, Kianoosh T, Tanyolac MB. Genetic diversity and population structure of common bean (*Phaseolus vulgaris* L.) accessions through retrotransposon-based interprimer binding sites (iPBSs) markers. Turk J Agric For. 2015; 39(6): 940–948.

[32] Nemli S, Aşçioğul TK, Ateş D, Eşiyok D, and Tanyolac M.B. Diversity and genetic analysis through DArTseq in common bean (*Phaseolus vulgaris* L.) germplasm from Turkey. Turk J Agric For. 2017; 41(5):389–404.

[33] IBPGR. (1982). International Board for Plant Genetic Resources Descriptors of Phaseolus vulgaris. Roma, Italy

[34] Doyle J. J. (1990). Isolation of plant DNA from fresh tissue. Focus, 12, 13–15.

[35] Baloch FS, Alsaleh A, Andeden EE, Hatipoğlu R, Nachit M, Özkan H. High levels of segregation distortion in the molecular linkage map of bread wheat representing the West Asia and North Africa region. Turk J Agric For. 2016; 40(3): 352–364.

[36] Elshire RJ, Glaubitz JC, Sun Q, Poland JA, Kawamoto K, Buckler ES, et al A robust, simple genotyping-by-sequencing (GBS) approach for high diversity species. PloS one. 2011; 6(5):e19379: 10.1371/journal.pone.0019379 PMC3087801 21573248

[37] Kilian A, Wenzl P, Huttner E, Carling J, Xia L, Blois H, et al Diversity arrays technology: a generic genome profiling technology on open platforms. Data Production and Analysis in Population Genomics: M&Ps. 2012:67–89. 10.1007/978-1-61779-870-2_5 22665276

[38] Raman H, Raman R, Kilian A, Detering F, Carling J, Coombes N, et al Genome-wide delineation of natural variation for pod shatter resistance in Brassica napus. PLoS One. 2014;9(7): e101673: 10.1371/journal.pone.0101673 PMC4090071 25006804

[39] H, Vikram P, Singh RP, Kilian A, Carling J, Song J, Burgueno-Ferreira JA, et al A high density GBS map of bread wheat and its application for dissecting complex disease resistance traits. BMC genomics. 2015;16(1):216. 25887001 10.1186/s12864-015-1424-5 PMC4381402

[40] R Core Team. (2013). R: A Language and Environment for Statistical Computing. R Foundation for Statistical Computing, Vienna, Austria. ISBN 3-900051-07-0, Available at: http://www.R-project.org/.

[41] Goudet J, Raymond M, de Meeüs T, Rousset F. Testing differentiation in diploid populations. Genetics. 1996; 144(4): 1933–1940. 8978076 10.1093/genetics/144.4.1933 PMC1207740

[42] Yang RC. Estimating hierarchical f‐statistics. Evolution. 1998;52(4): 950–956. 10.1111/j.1558-5646.1998.tb01824.x 28565205

[43] VanRaden PM (2008) Efficient methods to compute genomic predictions. J Dairy Sci. 91:4414–23. 10.3168/jds.2007-0980 18946147

[44] Letunic I, Bork P. Interactive tree of life (iTOL) v3: an online tool for the display and annotation of phylogenetic and other trees. Nucleic Acids Res. 2016;44: 242–245. 10.1093/nar/gkw290 PMC4987883 27095192

[45] Pritchard JK, Stephens M, Donnelly P. Inference of population structure using multilocus genotype data. Genetics. 2000;155(2): 945–959. 10835412 10.1093/genetics/155.2.945 PMC1461096

[46] Evanno G, Regnaut S, Goudet J. Detecting the number of clusters of individuals using the software STRUCTURE: a simulation study. Mol Ecol. 2005;14(8):2611–2620. 10.1111/j.1365-294X.2005.02553.x 15969739

[47] Bouchet S, Pot D, Deu M, Rami JF, Billot C, Perrier X, et al Genetic structure, linkage disequilibrium and signature of selection in sorghum: lessons from physically anchored DArT markers. PLoS ONE. 2012: 7(3), e33470 10.1371/journal.pone.0033470 22428056 PMC3302775

[48] Newell MA, Cook D, Hofmann H, Jannink JL. An algorithm for deciding the number of clusters and validation using simulated data with application to exploring crop population structure. Ann. App. Stat.2013: 7:1898–1916.

[49] Habyarimana E. Genomic prediction for yield improvement and safeguarding of genetic diversity in CIMMYT spring wheat (*Triticum aestivum* L.). Aust. J. Crop Sci. 2016:10: 127–136.

[50] Andeden EE, Baloch FS, Çakır E, Toklu F, Özkan H. Development, characterization and mapping of microsatellite markers for lentil (*Lens culinaris* Medik.). Plant Breed. 2015;134(5): 589–598.

[51] Nadeem MA, Nawaz MA, Shahid MQ, Doğan Y, Comertpay G, Yıldız M, et al DNA molecular markers in plant breeding: current status and recent advancements in genomic selection and genome editing. Biotechnol. Biotechnol. Equip. 2018:32:261–285.

[52] Nawaz MA, Sadia B, Awan FS, Zia MA, Khan IA. Genetic diversity in hyper glucose oxidase producing aspergillus niger UAF mutants by using molecular markers. Int. J. Agric. Biol. 2013;15: 362–366.

[53] Angioi SA, Rau D, Attene G, Nanni L, Bellucci E, Logozzo G, et al Beans in Europe: origin and structure of the European landraces of *Phaseolus vulgaris* L. Theor Appl Genet. 2010;121(5): 829–843. 10.1007/s00122-010-1353-2 20490446

[54] Habyarimana E, Parisi B, Mandolino G. Genomic prediction for yields, processing and nutritional quality traits in cultivated potato (*Solanum tuberosum* L.). Plant Breed. 2017: 136, 245–252. 10.1111/pbr.12461

[55] Cichy KA, Fernandez A, Kilian A, Kelly JD, Galeano CH, Shaw S, et al QTL analysis of canning quality and color retention in black beans (*Phaseolus vulgaris* L.). Mol Breed. 2014;33(1): 139–54.

[56] Valdisser PA, Pereira WJ, Almeida Filho JE, Müller BS, Coelho GR, de Menezes IP, et al In-depth genome characterization of a Brazilian common bean core collection using DArTseq high-density SNP genotyping. BMC genomics. 2017;18(1): 10.1186/s12864-017-3805-4 PMC5450071 28558696

[57] Valdisser PA, Pappas GJ Jr, de Menezes IP, Müller BS, Pereira WJ, Narciso MG, et al SNP discovery in common bean by restriction-associated DNA (RAD) sequencing for genetic diversity and population structure analysis. Mol Genet Genomics. 2016;291(3): 1277–91. 10.1007/s00438-016-1182-3 26932372

[58] Mukeshimana G, Butare L, Cregan PB, Blair MW, Kelly JD. Quantitative trait loci associated with drought tolerance in common bean. Crop Sci. 2014;54(3): 923–938.

[59] Schröder S, Mamidi S, Lee R, McKain MR, McClean PE, Osorno JM. Optimization of genotyping by sequencing (GBS) data in common bean (*Phaseolus vulgaris* L.). Mol Breed. 2016;36(1): 10.1007/s11032-015-0431-1

[60] Blair MW, Cortés AJ, Penmetsa RV, Farmer A, Carrasquilla-Garcia N, Cook DR. A high-throughput SNP marker system for parental polymorphism screening, and diversity analysis in common bean (*Phaseolus vulgaris* L.). Theor Appl Genet. 2013;126(2): 535–548. 10.1007/s00122-012-1999-z 23124389

[61] Wani AB, Bhat MA, Husaini AM, Sidiqi I. Screening of important bean genotypes/collections for resistance against Common Bean Mosaic Virus using molecular markers. J Pharmacogn Phytochem. 2017;6(4): 343–347.

[62] Kumar V, Sharma S, Kero S, Sharma S, Sharma AK, Kumar M, et al Assessment of genetic diversity in common bean (*Phaseolus vulgaris* L.) germplasm using amplified fragment length polymorphism (AFLP). Sci Hort. 2008;116(2):138–143.

[63] Burle ML, Fonseca JR, Kami JA, Gepts P. Microsatellite diversity and genetic structure among common bean (*Phaseolus vulgaris* L.) landraces in Brazil, a secondary center of diversity. Theor Appl Genet. 2010;121(5): 801–13. 10.1007/s00122-010-1350-5 20502861 PMC2940433

[64] Blair MW, Díaz LM, Buendía HF, Duque MC. Genetic diversity, seed size associations and population structure of a core collection of common beans (*Phaseolus vulgaris* L.). Theor Appl Genet. 2009;119(6): 955–72. 10.1007/s00122-009-1064-8 19688198

[65] McClean PE, Terpstra J, McConnell M, White C, Lee R, Mamidi S. Population structure and genetic differentiation among the USDA common bean (*Phaseolus vulgaris* L.) core collection. Genet Resour Crop Evol.2012: 59: 499–515.

[66] Rodriguez M, Rau D, Bitocchi E, Bellucci E, Biagetti E, Carboni A, et al Landscape genetics, adaptive diversity and population structure in *Phaseolus vulgaris*. New Phytol. 2016;209(4): 1781–94. 10.1111/nph.13713 26526745

[67] Govindaraj M, Vetriventhan M, Srinivasan M. Importance of genetic diversity assessment in crop plants and its recent advances: an overview of its analytical perspectives. Genet Res Int. 2015: 10.1155/2015/431487 25874132 PMC4383386

[68] Lombardi M, Materne M, Cogan NO, Rodda M, Daetwyler HD, Slater AT, et al Assessment of genetic variation within a global collection of lentil (*Lens culinaris Medik*.) cultivars and landraces using SNP markers. BMC genetics. 2014 15(1):150. 25540077 10.1186/s12863-014-0150-3 PMC4300608

[69] Ceylan A, Öcal N, Akbulut M. Genetic diversity among the Turkish common bean cultivars (*Phaseolus vulgaris* L.) as assessed by SRAP, POGP and cpSSR markers. Biochemical Systematics and Ecology. 2014 1;54:219–29.

[70] Carović-Stanko K, Liber Z, Vidak M, Barešić A, Grdiša M, Lazarević B, et al Genetic Diversity of Croatian Common Bean Landraces. Front Plant Sci. 2017: 10.3389/fpls.2017.00604 28473842 PMC5397504

